# Continuous Protein Sensing Using Fast-Dissociating Antibody Fragments in Competition-Based Biosensing by Particle Motion

**DOI:** 10.1021/acssensors.4c03637

**Published:** 2025-03-24

**Authors:** Claire M. S. Michielsen, Yu-Ting Lin, Junhong Yan, Arthur M. de Jong, Menno W. J. Prins

**Affiliations:** † Department of Biomedical Engineering, Eindhoven University of Technology, Eindhoven 5612 AE, the Netherlands; ‡ Department of Applied Physics, Eindhoven University of Technology, Eindhoven 5612 AE, the Netherlands; § Institute for Complex Molecular Systems (ICMS), Eindhoven University of Technology, Eindhoven 5612 AE, the Netherlands; ∥ Helia Biomonitoring, Eindhoven 5612 AE, the Netherlands

**Keywords:** continuous protein sensing, antibody fragments, binding kinetics, competition biosensor, biosensing by particle motion

## Abstract

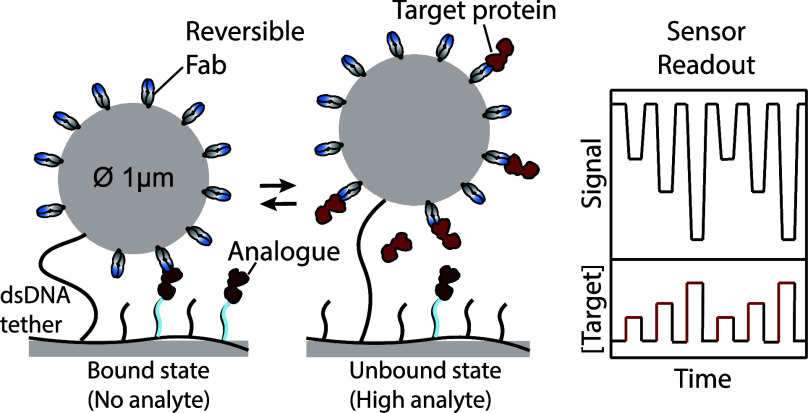

Sensing technologies for the continuous monitoring of protein concentrations are important for understanding time-dependent behaviors of biological systems and for controlling bioprocesses. We present a continuous sensing methodology based on tethered particle motion (t-BPM) that utilizes fast-dissociating antibody fragments (Fabs) for continuous protein monitoring. A competition-based t-BPM sensor was developed and characterized utilizing custom-made Fabs. The sensing concept was demonstrated for lactoferrin, an 80 kDa iron-binding glycoprotein that is part of the innate immune response. Thirteen Fabs were compared using free particle motion sensing as well as surface plasmon resonance, of which six Fabs showed rapid association and dissociation. The integration of the Fabs into the t-BPM sensor enabled nanomolar lactoferrin detection in both buffer solutions and milk matrices over tens of hours. This work demonstrates how continuous protein sensing can be realized using fast-dissociating antibodies in a competitive sensor format.

Proteins are biomolecules that regulate and reflect the dynamic properties of biological systems. To gain understanding and control over time-dependent bioprocesses, it would be highly valuable to have sensors available that can track the dynamics of specific protein concentrations over long time spans. The time-resolved measurements could benefit areas such as fundamental biological research, patient monitoring^1−3^ and industrial bioprocessing.^4,5^

A generic methodology for detecting proteins is to use affinity-based interactions, where specific binder molecules such as antibodies or aptamers, selectively recognize and attach to target proteins. Commercial antibodies are typically developed to bind strongly to their targets, characterized by high binding affinity and low dissociation rate constants (low *k*_off_). These properties make the binders suitable for bioanalysis techniques such as immunoassays, immunohistochemistry, and Western blots, which have long incubation times, multiple washing cycles and endpoint readouts. However, the use of high-affinity binding can result in slow reversibility or no reversibility at all, which can severely limit the ability to achieve continuous biosensing. In contrast, binder molecules with high dissociation rate constants are promising, as they can enable spontaneous sensor reversibility and facilitate monitoring of increases and decreases in protein concentrations with good time resolution.^6−8^

Biosensing by tethered Particle Motion (t-BPM) is an affinity-based continuous sensing technology with single-molecule resolution that relies on reversible molecular binding.^9^ In t-BPM, biofunctionalized particles are tethered to a biofunctionalized substrate, and interactions between the particles and substrate are modulated by the concentration of the analyte in solution. The t-BPM sensing technology has been applied to monitor small molecules, using antibody functionalized particles and a competing analyte-analogue coupled to the substrate.^10−12^ Protein sensing was explored for thrombin, using aptamers as binder molecules,^9^ with limited reversibility and a relatively short operational lifetime.

In this study, we investigate the design of a continuous sensor for reversible protein measurements over long time spans. The sensor utilizes fast-dissociating antibody fragments (Fabs) in a competition-based t-BPM sensor format. The methodology is demonstrated for lactoferrin, an 80 kDa iron-binding glycoprotein that supports the immune system and is present in secretory fluids.^13^ Lactoferrin is extracted from bovine milk and used in infant nutrition and as a dietary supplement.^14^ Continuous monitoring sensors with a response time of around 10 minutes are anticipated to enable the real-time control of time dependencies in industrial manufacturing processes. In this paper, we report a study of the binding kinetics of custom-made antibody fragments using surface plasmon resonance and BPM. Fabs with suitable kinetic properties were implemented in a competition-based t-BPM sensor and the analytical performances were compared. Finally, continuous lactoferrin monitoring was demonstrated over long time spans (tens of hours) in both buffer solutions and milk.

## Results and Discussion

### Antibody Development and Characterization for Continuous Biosensing

**Figure 1:**
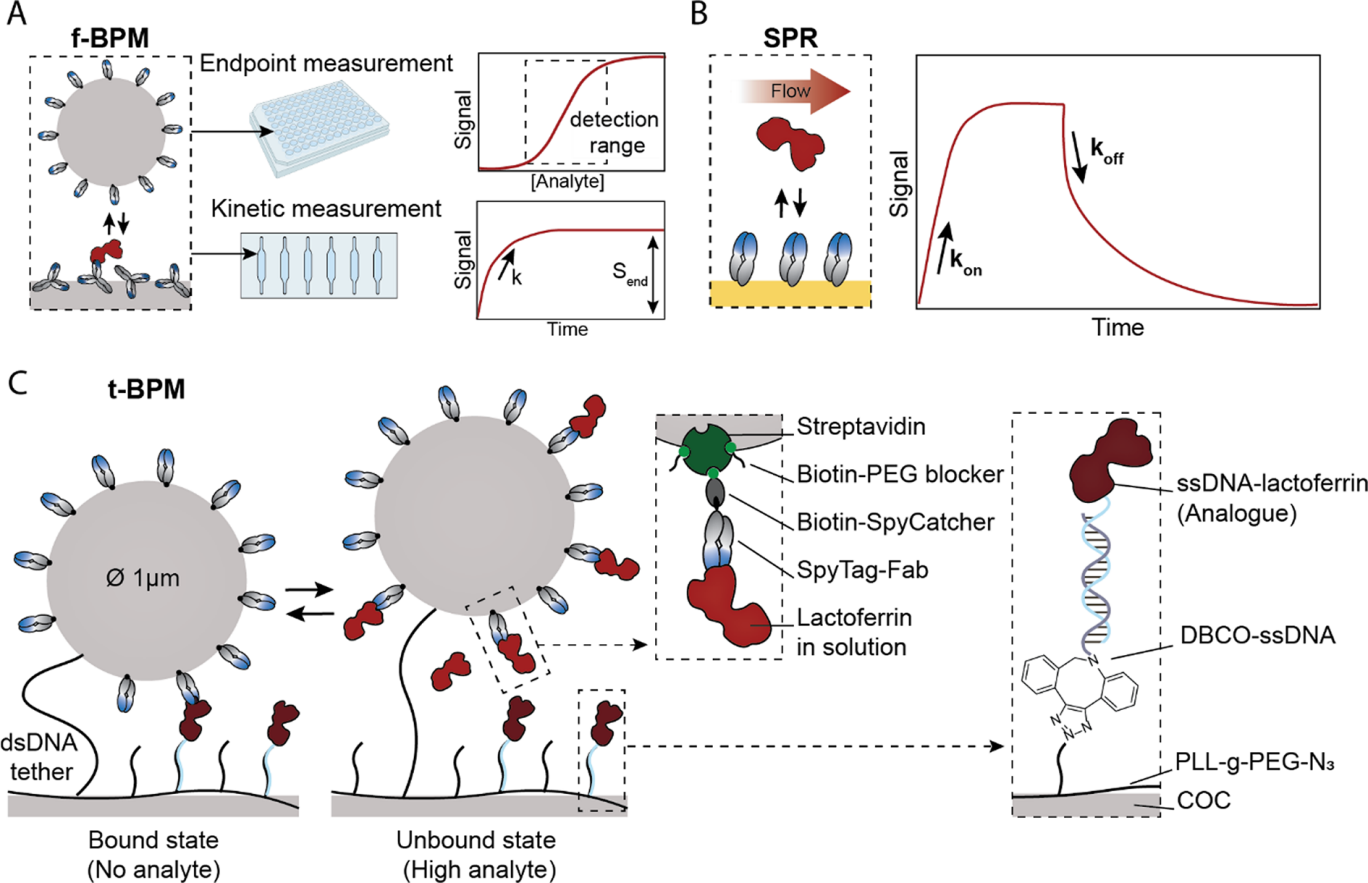
Experimental design for mapping Fab kinetics and the implementation of Fabs in a competitive t-BPM protein sensor. (A) Screening of Fabs using Biosensing by free Particle Motion (f-BPM). The Fabs were immobilized on particles and anti-lactoferrin polyclonal antibodies on the substrate. The detection range was determined using endpoint measurements in a 96-well plate, and kinetic measurements were performed in flow cells to determine the characteristic rate of signal increase (*k*) and the final signal (*S*_end_). (B) Mapping binder kinetics using Surface Plasmon Resonance (SPR). The association (*k*_on_) and dissociation (*k*_off_) rates were measured of the different Fabs immobilized on a gold-coated SPR chip. (C) Molecular design of the competition-based continuous protein sensor based on tethered particle motion (t-BPM). A COC (cyclic olefin copolymer) sensor surface was coated with a low-fouling PLL-*g*-PEG polymer, to which the analyte competitor (analogue) was coupled.^15^ Double-stranded DNA was attached to the polymer, which tethers the particles to the sensor surface. The particles were functionalized with Fabs that reversibly bind to the analyte and the immobilized analogue. Bound and unbound states of the particles were detected using video microscopy and particle tracking software.^16^

Fast-dissociating antibodies can be developed using phage display methodologies and synthetic antibody libraries, which allow for the selection of low-affinity variants.^6,17−19^ Custom-made Fabs were ordered from Bio-Rad and produced using a human combinatorial antibody library (HuCAL technology) and phage-display selection.^17,20^ Thirteen candidates with potentially fast-dissociating kinetics, based on a *k*_off_ ranking, were received from Bio-Rad for further investigations in this study. As a first step, the Fabs were studied using the BPM sensing method with free particles, known as free-BPM (f-BPM),^21^ illustrated in Figure 1A. In the sandwich-based f-BPM experiment, biofunctionalized particles (1 μm in diameter) move randomly over a biofunctionalized substrate due to Brownian motion. When a target protein is present in solution, the binders on the particle and substrate form a sandwich bond, restricting the motion of the particle, referred to as a bound state. In BPM, the motion of individual particles is measured using video microscopy and particle tracking software (Supporting Information Figure S1).^16^ The primary readout parameter in this study is the bound fraction, defined as the ratio of the population of bound states to the total number of states observed within the measurement time for all tracked particles in the field of view.

In the f-BPM study, particles were prepared with the different Fabs and an anti-lactoferrin polyclonal antibody was physisorbed on the substrate (Figure 1A). The polyclonal antibodies, which have been used in a previous study,^22^ irreversibly bind to different epitopes of lactoferrin, enabling the screening of the different Fabs on the particles. Measurements were done in a 96-well plate using varying lactoferrin concentrations to generate dose–response curves and to determine the detection range. Additionally, kinetic measurements were conducted using flow cells to assess the signal time dependency for each Fab. To gain more insights into the binding kinetics of the Fabs, Surface Plasmon Resonance (SPR) measurements were used (Figure 1B). In SPR, molecular interactions are monitored in real-time in order to determine the binding kinetics (*k*_on_ and *k*_off_) of the Fabs immobilized on a gold surface.

After mapping the binding kinetics using f-BPM and SPR, the most suitable Fabs were integrated into the competition-based t-BPM sensor (Figure 1C).^10−12,15^ The sensor design uses streptavidin-coated particles functionalized with Fabs that are tethered to the sensor surface via a double-stranded DNA tether (dsDNA, 221 bp, ∼75 nm). The dsDNA tether is modified with biotin and dibenzocyclooctyne (DBCO) at each end (Supporting Information Table S1). The COC (cyclic olefin copolymer) sensor surface is coated with a low-fouling polymer mixture of poly(l-lysine)-grafted-poly(ethylene glycol) (PLL-*g*-PEG) and PLL-*g*-PEG-azide. The azide moieties of the polymer are functionalized with DBCO-modified molecules, including the dsDNA tether and single-stranded DNA (ssDNA), referred to as docking DNA (Supporting Information Table S1). The streptavidin-coated particles functionalized with biotinylated binders are partly blocked using biotin-PEG (1 kDa) to prevent multitethering when the particles are added to the DNA functionalized substrate.^15^ Complementary ssDNA-lactoferrin conjugates are hybridized to the docking DNA, functioning as the analogue in the competition assay. In the absence of analyte, the Fabs on the particle have a high probability to bind to analogue molecules on the sensor surface, leading to frequent particle binding. When free lactoferrin is present, the Fabs on the particle bind to the lactoferrin molecules in solution, increasing the probability that particles remain in an unbound state.

### Screening of Fabs Using Biosensing by Free Particle Motion (f-BPM)

**Figure 2:**
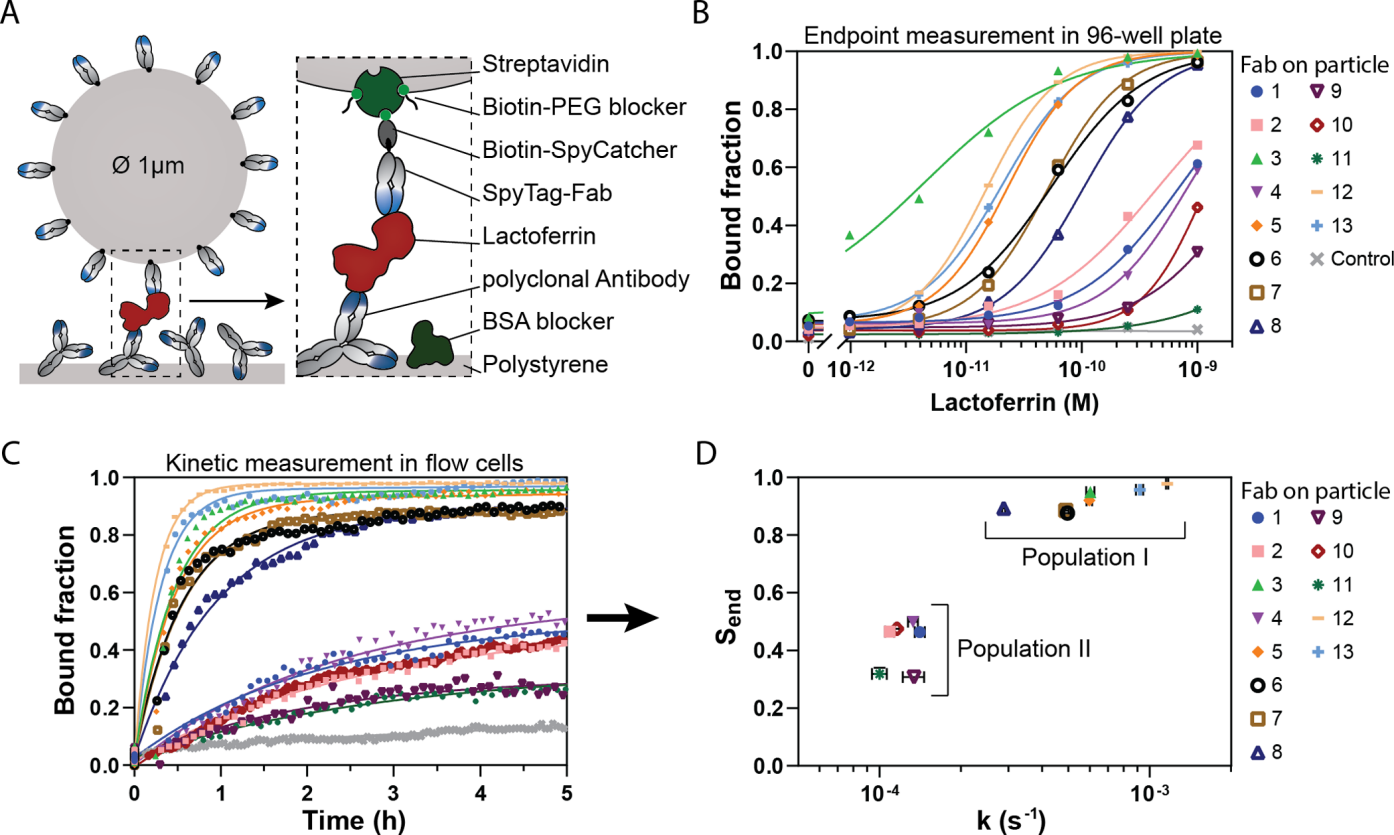
Screening of Fabs using Biosensing by free Particle Motion (f-BPM). (A) Schematic representation of the sandwich f-BPM immunosensor, with polyclonal antibodies physisorbed on the sensor surface and biotin-Fabs on streptavidin-coated particles (1 μm diameter). The sensor surface was blocked with bovine serum albumin (BSA) and the remaining streptavidin binding sites were blocked with biotin-PEG (1 kDa). In the presence of the analyte, lactoferrin, a sandwich bond forms, restricting the motion of the particle. Details on the sensor readout are provided in Supporting Information Figure S1. (B) Dose–response curves for all 13 Fabs, each represented by a different color and symbol, measured in a 96-well plate. Particles without Fabs were used as a negative control (gray cross). Every data point is a bound fraction value measured after approximately 10 h of incubation (Supporting Information Figure S4, series 10). The solid lines are guides to the eye. (C) Time-dependent signal of the f-BPM sensor for each Fab, measured in a static flow cell after the addition of 500 pM lactoferrin. Data points are represented by symbols and the solid lines correspond to the fit using eq 1. The flow cells were monitored for 18 h, full curves are shown in Supporting Information Figure S6. (D) Final signal (*S*_end_; *y*-axis) and characteristic response rate (*k*; *x*-axis) for each Fab, extracted from the fits in panel C. Error bars (not always visible) represent the 95% confidence interval for the values derived from the fits.

Thirteen recombinant Fabs were screened using the f-BPM assay, with the molecular architecture of the setup illustrated in Figure 2A. The Fabs were engineered with a SpyTag, enabling site-specific conjugation of biotin via SpyCatcher-biotin (Supporting Information Figure S3).^23^ Biotin was used to immobilize the Fabs on the streptavidin-coated particles, and biotin-PEG (1 kDa) was used as a blocking agent. The polystyrene substrate was functionalized with polyclonal antibodies via physisorption, and the remaining surface was blocked with bovine serum albumin (BSA).

Endpoint measurements were performed in a 96-well plate to study the detection range of the Fabs (Figure 2B). All Fabs exhibited low background signals in the absence of lactoferrin and the bound fraction increases for increasing lactoferrin concentrations. The Fabs can be categorized into two populations based on their detection ranges: population I (Fab 3, 5, 6, 7, 8, 12, and 13) displayed a low picomolar detection range, while population II (Fab 1, 2, 4, 9, 10, and 11) demonstrated a higher detection range, spanning from high picomolar to low nanomolar concentrations. Additional measurements with higher detection ranges are detailed in Supporting Information Figure S5.

Notably, Fab 3 was able to detect the lowest lactoferrin concentrations but appeared less sensitive due to a shallower slope in the dose–response curve compared to the other Fabs in Population I. Looking at the dose–response curves over time, a shift toward a shallower slope was revealed in the curve for Fab 3, a phenomenon that was significantly less pronounced for the other Fabs within this group (Supporting Information Figure S4). This observation leads to the following hypothesis: when particles bind irreversibly to the substrate, they continue to search for and eventually find a lactoferrin molecule, causing the signal to increase over time until all particles are bound. In contrast, when particles bind reversibly, an equilibrium is established, resulting in less drift in the signal. Thus, it is suggested that Fab 3 may be less reversible in its binding compared to the other Fabs in Population I.

The response over time was further investigated through kinetic measurements in flow cells with 500 pM lactoferrin. The distinction between the two populations became even clearer when examining the signal response over time (Figure 2C). Fabs in Population I, which exhibit picomolar detection ranges, showed rapid signal increases with substantial signal changes. In contrast, Fabs in Population II, with high picomolar to nanomolar detection ranges, displayed much slower signal increases and significantly lower overall signal changes. To quantify these differences, the kinetic association curves were fitted with an exponential plus linear model:
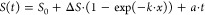
1with *S*_0_ the signal at *t* = 0 of the measurement, and ΔS the amplitude of the exponential response. The final signal (plateau) of the exponential part of the association curve is defined as *S*_end_ = *S*_0_ + Δ*S*. *k* is the characteristic response rate and *a* represents the slope of the linear part of the signal that is attributed to a slow increase of the background. Data from the 15-h measurement is shown in Supporting Information Figure S6. Figure 2D shows the final signal (*S*_end_) and characteristic response rate (*k*) for each Fab. The characteristic response time τ, defined as τ = 1/*k*, varied significantly between the two populations: Fabs in Population I had response times ranging from 15 to 60 min, while those in Population II exhibited much longer response times, ranging from 2 to 3 h (Supporting Information Table S2). In summary, Fabs from Population I demonstrated both faster response times and significantly higher signal amplitudes, resulting in greater sensitivity to lower concentrations compared to the Fabs from Population II.

These findings align with the observations of the 96-well plate measurements. The higher detection range of Population II can be attributed to slower association rates of the Fabs compared to those in Population I.

### Mapping Binder Kinetics Using Surface Plasmon Resonance (SPR)

**Figure 3:**
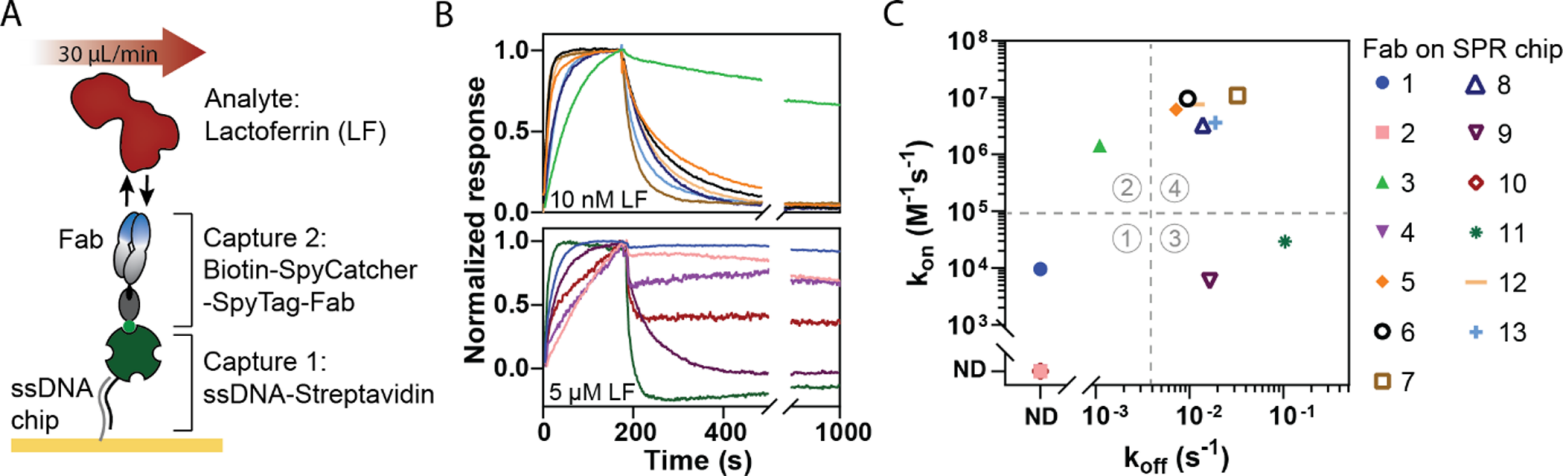
Mapping binder kinetics using surface plasmon resonance (SPR). (A) Schematic representation of the molecular composition of the SPR sensor. A DNA-coated chip was functionalized with ssDNA-streptavidin and Biotin-SpyCatcher-SpyTag-Fab complexes. The analyte solution (lactoferrin diluted in PBS with 500 mM NaCl and 0.005% P20) was flown through the flow cell at a rate of 30 μL/min. (B) Thirteen different Fabs (1–13) were each immobilized on the chip. Association was measured for 180s using either 10 nM or 5 μM lactoferrin. Dissociation was measured for 15 min in the absence of lactoferrin in the running buffer. (C) Association (*k*_on_; *y*-axis) and dissociation (*k*_off_; *x*-axis) rates were determined for all Fabs. Fitted data is presented in Supporting Information Figure S7 and Table S3. For some Fabs (2, 4, 10), the rates could not be determined because of unstable signals resulting from nonspecific interactions at high lactoferrin concentrations (indicated with ND, not determined).

To gain more insights into the binding kinetics of the Fabs, the interaction between lactoferrin and immobilized Fabs was further analyzed using SPR. Figure 3A illustrates the molecular composition of the SPR sensor. A single-stranded DNA (ssDNA) coated chip was used in combination with a complementary ssDNA-streptavidin conjugate, facilitating the immobilization of biotinylated Fabs via the biotin–streptavidin interaction. The ssDNA-streptavidin complex can be removed using a regeneration buffer, allowing the chip to be reused for all Fabs.

The binding kinetics of the Fabs from Population I were measured using 10 nM lactoferrin, while those from Population II were measured with 5 μM lactoferrin (Figure 3B). All Fabs from Population I exhibited complete dissociation within 3–10 min, with the exception of Fab 3, which showed a much slower dissociation rate. Measuring the Fabs from Population II posed more challenges due to the high protein concentration, which led to increased nonspecific interactions. Specifically, Fab 2, 4, and 10 produced unstable signals with no clear association or dissociation patterns. However, clear association curves were observed for Fab 1, 9, and 11. Of these, Fab 1 showed no dissociation, Fab 9 exhibited complete dissociation, and Fab 11 demonstrated very rapid dissociation.

The association and dissociation curves for both populations were fitted with a single exponential rate model to extract the association rates (*k*_on_) and dissociation rates (*k*_off_).The extracted values are displayed in Figure 3C and the fitted curves in Supporting Information Figure S7. Based on their binding kinetics, the Fabs were categorized into four categories: (1) slow association and slow dissociation, (2) fast association and slow dissociation, (3) slow association and fast dissociation, and (4) fast association and fast dissociation. Most Fabs from Population I exhibited fast association and fast dissociation, placing them in Category 4, except for Fab 3, which fell into Category 1 due to its slow dissociation. Fabs 5, 6, 7, 8, 12, and 13 were all categorized under Category 4. In Population II, the Fabs generally showed slow association rates. Among those successfully measured by SPR, Fab 1 was classified into Category 1, while Fabs 9 and 11 were placed in Category 3. In conclusion, six Fabs (5, 6, 7, 8, 12 and 13) demonstrated the fast association and dissociation rates necessary for continuous protein sensing. In the next section, these Fabs will be studied for implementation in a t-BPM competition sensor.

### Continuous Lactoferrin Sensing Using Biosensing by Tethered Particle Motion (t-BPM)

**Figure 4:**
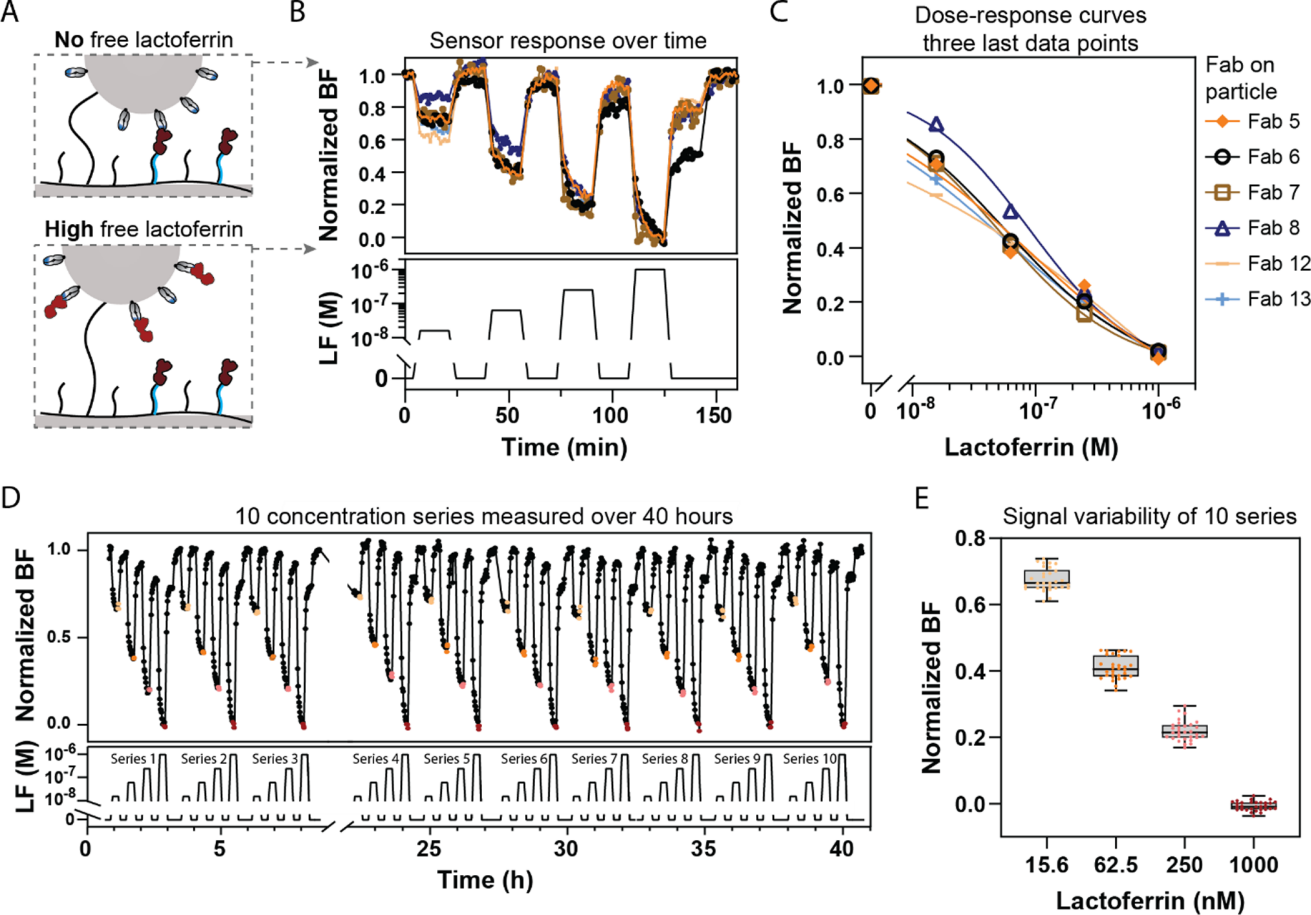
Continuous lactoferrin sensing using Biosensing by tethered Particle Motion (t-BPM). (A) Schematic representation of a single particle in the t-BPM sensor. Particles were functionalized with Fabs and attached to the sensor surface via a dsDNA tether on a low-fouling polymer layer. ssDNA-lactoferrin conjugates (analogues) were hybridized to the ssDNA on the polymer. In the absence of free lactoferrin (top), the particle binds to the analogue molecules on the substrate. When free lactoferrin is present (bottom), the Fabs on the particle bind to the free analyte, preventing interactions with the analogue molecules on the substrate. (B) t-BPM sensor response as a function of time under varying lactoferrin concentrations, for Fab 5, 6, 7, 8, 12, and 13. The top panel shows the normalized sensor response. The bottom panel depicts the time profile of the lactoferrin concentration (0, 15.6, 62.5, 250, and 1000 nM lactoferrin in PBS with 500 mM NaCl) supplied into the flow cell. Samples were flushed in at a flow rate of 100 μL/min for 1 min. The bound fraction was measured in the absence of flow with 1-min intervals over 15 min. (C) Dose–response curves for the six Fabs, derived from the data in panel B. The symbols represent the average of the final three data points measured for each concentration. The solid lines represent sigmoidal dose–response fits for each Fab. (D) Continuous measurement of 10 series of four lactoferrin concentrations using a t-BPM sensor with Fab 13 on the particles, measured over a total period of 40 h. The top panel shows the normalized signal as a function of time and the bottom panel indicates the supplied lactoferrin concentrations. (E) Variability of the final signal (colored data points in panel D) determined for each lactoferrin concentration. Colored dots represent the measured data points. The boxes illustrate the distribution of data points: the whiskers represent the full range (minimum to maximum), the horizontal line within the box indicates the median, and the box itself encompasses the interquartile range, which represents 50% of the data points.

The Fabs with fast association and dissociation rates were evaluated in a competition-based t-BPM sensor. Particles functionalized with the Fabs were tethered to a low-fouling polymer layer, and ssDNA-lactoferrin conjugates were hybridized to ssDNA coupled to the polymer layer (Figure 4A). In the absence of free lactoferrin, the Fabs can bind to the immobilized ssDNA-lactoferrin conjugates, resulting in a high bound fraction. When free lactoferrin is added, the bound fraction decreases due to competition between free and immobilized molecules for binding to the Fabs.

Four different concentrations of lactoferrin were tested, with a wash step performed after each concentration measurement to assess the binding reversibility (Figure 4B; multiple series are shown in Supporting Information Figure S9). All six Fabs exhibited a high bound fraction when free lactoferrin was absent, and a decrease in bound fraction when free lactoferrin was added. This confirmed that all six Fabs could bind to the immobilized lactoferrin on the substrate and dissociate in the presence of free lactoferrin. The responses of all Fabs were consistent, displaying a similar pattern across all samples. After exposure of the sensor to low nanomolar lactoferrin concentrations, the supply of a blank sample caused the signal to return to the baseline (normalized bound fraction goes to 1). However, after exposure to higher lactoferrin concentrations, a single supply of the blank solution did not bring the signal back to the baseline and an additional wash step was required. This indicates that higher lactoferrin concentrations require more extensive washing due to the larger number of molecules that need to be removed from the measurement chamber.

Some differences could be observed between the Fabs. Fab 6 (in black) showed the largest decrease in baseline level with increasing lactoferrin concentrations, suggesting a slower dissociation rate compared to the other Fabs (Figure 4B). Additionally, Fab 8 showed a smaller signal change at low lactoferrin concentrations, indicating a lower sensitivity, likely due to a lower association rate, as its reversibility was comparable to the other Fabs. Figure 4C presents the dose–response curves for each Fab, showing that the detection window is in the nanomolar range and very similar across all Fabs, with the exception of Fab 8, which demonstrated smaller signal changes at low nanomolar concentrations.

In Figure 4B, each sample was measured using 15 consecutive 1-min recordings of the same sample without any flow. Over the course of the measurements, a time-dependent signal was consistently observed for all samples, regardless of the analyte concentration (Supporting Information Section 6.4). The time dependency may reflect the dissociation rate constants of the Fabs, which, based on surface plasmon resonance (SPR) data, have a characteristic dissociation time on the order of a few minutes. Alternatively, the time dependency may relate to incomplete fluid exchange in the measurement chamber, which can lead to slow diffusive equilibration, creating time-dependent concentration gradients.

Figure 4D presents 10 series of four lactoferrin concentrations, alternated with blank samples, measured in a t-BPM sensor with Fab 13 over a 40-h period. To correct for the drift in the signal of the competition sensor, the data were normalized using linear interpolation between the highest and lowest signal points in the competition sensor: the highest signal corresponds to the baseline measurement (signal in the absence of analyte), and the lowest signal corresponds to the background measurement (signal with a known high concentration of analyte). These points were used to create a linear reference line. Each data point in the time series was then adjusted according to its position relative to the reference line, effectively compensating for signal drift in the sensor (Supporting Information Figure S10). After normalization, a stable and reproducible signal was observed over the 40 h. The signal variabilities of the final three data points for each concentration over 40 h are illustrated in Figure 4E.

In conclusion, the six selected Fabs could be implemented in a competitive t-BPM sensor and demonstrated continuous lactoferrin sensing with consistent and reproducible results. The sensors detected changes in bound fraction with varying lactoferrin concentrations, confirming the ability of the Fabs to bind and dissociate in response to free lactoferrin. The recordings over extended periods underscore the suitability of the sensor for long-term measurements.

### Signal and Concentration Variability in Buffer and Milk Samples

**Figure 5:**
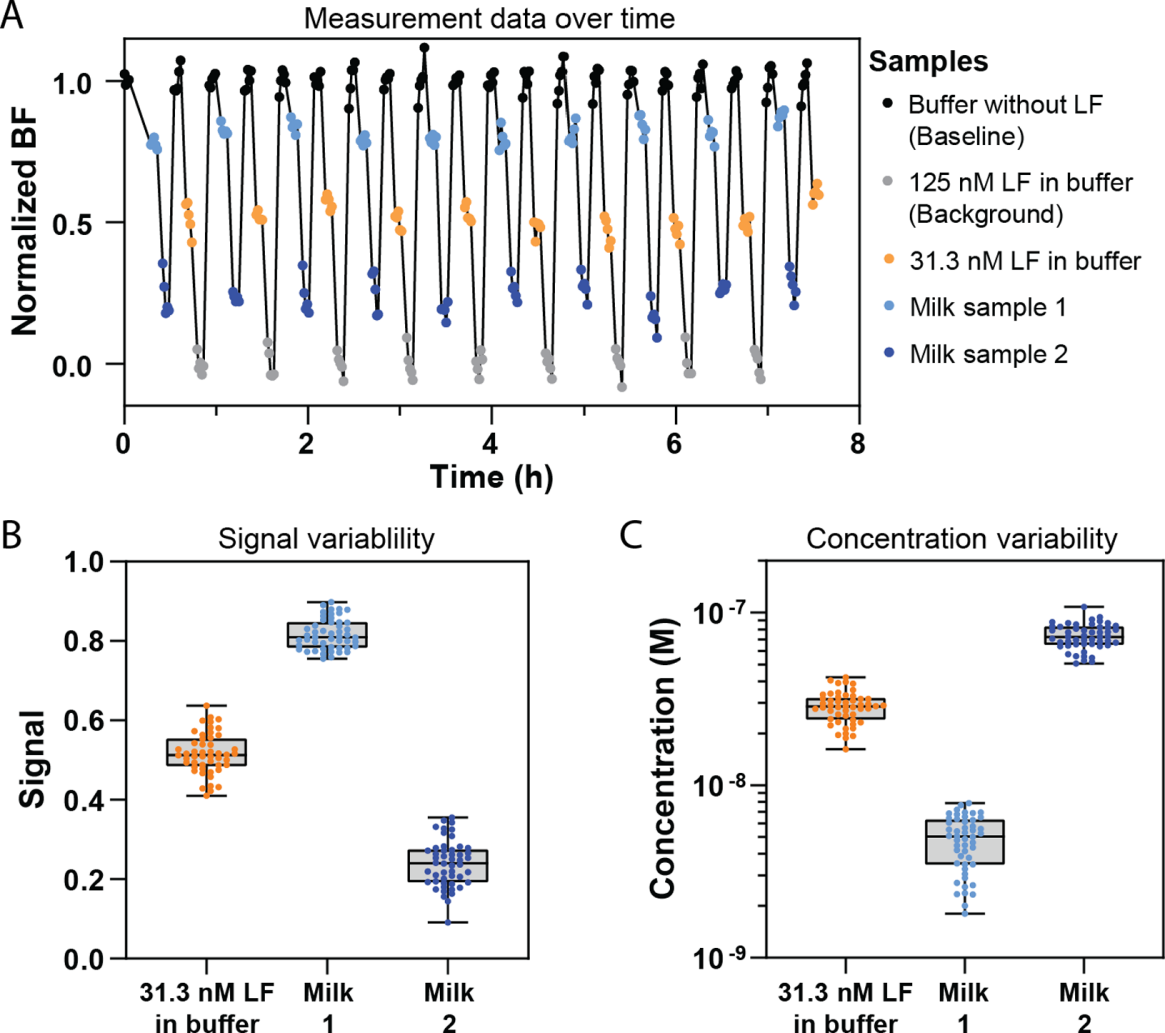
Signal and concentration variability of lactoferrin measurements in buffer and milk samples using the competition-based t-BPM sensor. (A) Measurement data as a function of time for different samples using Fab 13. Baseline samples (black; 0 nM LF) and background samples (gray; 125 nM LF) were used to normalize the sensor response. A buffer sample with 31.3 nM LF (orange) and two milk samples (light and dark blue) were measured repeatedly over time. The milk samples were centrifuged for 10 min at 10.000 rpm and diluted 10×. The bound fraction was measured in the absence of flow with 1-min intervals over a period of 5 min. (B) Signal variability of all bound fraction measurements for three samples (31.3 nM LF in buffer, milk 1, milk 2). (C) Concentration variability of all measurements on the three samples. The concentration values were derived from the calibration curve shown in Supporting Information Figure S11C. The boxes illustrate the distribution of the data points: the whiskers represent the full range (minimum to maximum), the horizontal line within the box indicates the median, and the box itself encompasses the interquartile range, which represents 50% of the data points.

The variabilities of the sensor signal and the determined lactoferrin concentrations were evaluated in both buffer and milk samples. The buffer samples were spiked with a known concentration of lactoferrin, while the milk samples naturally contained lactoferrin. For each sample, five consecutive 1-min measurements were done after sample addition, and this process was repeated across multiple series by resupplying the same samples to the sensor. The repeated measurements allow a quantification of variabilities of signal and concentration within a single sample addition (comparing the five consecutive measurements) and between different additions of the same sample (comparing the repeated series). Figure 5A shows a set of measurement data that was used to quantify the variabilities. Signal drift over time was corrected through normalization using linear interpolation between the highest (no lactoferrin) and lowest (125 nM lactoferrin) signal points, as described in the previous section. The raw data is presented in Supporting Information Figure S11.

Two types of samples were measured: buffer samples with a known concentration of lactoferrin and milk samples that were diluted 10-fold, after centrifugation for 10 min to remove any aggregates that could disturb the measurement. Data normalization was carried out using the highest concentration buffer sample, resulting in three samples for analyzing the variabilities: a buffer sample with 31.3 nM lactoferrin and two milk samples. Figure 5B illustrates the signal variabilities for these three samples. Variations were observed across the five consecutive measurements and between the different series. The measured variabilities can have several origins, including the BPM measurement itself, biomolecular processes such as nonspecific binding, and variabilities in fluidic exchanges.^24^ Variabilities can have random contributions (noise) as well as nonrandom contributions (drift). The bound fraction signals measured in Figures 4D and 5A show a drift component: consecutive 1-min measurements on a single sample can have a tendency to show increasing values or decreasing values. The drift component is attributed to incomplete fluid exchanges, which can cause time-dependent concentrations in the measurement chamber due to slow diffusive equilibration. Fluid exchange protocols can be optimized to reduce signal variabilities (Supporting Information Figure S13).

For each data point in Figure 5A, the lactoferrin concentration could be determined using the sensor calibration curve (Supporting Information Figure S11C). The corresponding concentration values are shown in Figure 5C. The buffer sample and milk sample 2 exhibit variabilities with coefficients of variation (i.e., standard deviation divided by the mean) of 20% and 17%, respectively. Milk sample 1 has a low lactoferrin concentration and resulted in a signal close to the baseline, which gave higher concentration variabilities because the dose–response curve is less steep at low concentrations (cf. Figure 4C and Supporting Information Figure S11C).

## Conclusions and Outlook

Reversible molecular binding is essential for the development of continuous protein biosensors. In this work, we characterized custom-made Fab binders and selected candidates with fast association and dissociation kinetics. Among the thirteen Fab binders analyzed using Biosensing by free Particle Motion (f-BPM) and Surface Plasmon Resonance (SPR), six demonstrated rapid kinetics suitable for continuous protein sensing. The selected Fabs were implemented in a competition-based t-BPM sensor, enabling continuous lactoferrin monitoring at nanomolar concentrations in buffer and milk samples, demonstrating reversible sensing over several tens of hours.

Continuous monitoring refers to the continuous collection of measurement data from a system of interest.^16^ One strategy to perform continuous monitoring is by taking consecutive samples as a function of time and by performing assays on every individual sample, consuming each time new assay reagents such as enzymes or antibodies.^25,26^ However, continuous reagent consumption makes it complicated to perform frequent measurements over long time spans. Another strategy is to use accumulative analyte capture on a biosensor with strong binding.^27^ However, the number of samples that can be measured on a sensor with accumulating analyte is limited by the saturation of the binder molecules in the sensor. Biosensors with reversible properties seem most suited for realizing long-term continuous monitoring. Biosensor reversibility can be obtained by applying a reset using chemical or physical conditions that break the bonds between analyte and binder molecules.^8,28,29^ However, regeneration methods involve an extra step in the sensor and may affect the long-term functionality of the binder molecules. Alternatively, biosensor reversibility can be achieved by using binder molecules with suitable thermodynamic properties. The binder molecules must have sufficiently strong binding properties to enable sensitive analyte measurements (low *K*_D_) while also exhibiting a sufficiently fast dissociation rate constant (high *k*_off_) to enable spontaneous sensor reversibility.

Continuous biomolecular sensing with spontaneous sensor reversibility, focusing on micromolar analyte concentrations and below, is being investigated using electrochemical aptamer-based methods,^30−33^ fluorescent molecular switches,^34−36^ and biosensing by particle motion.^9,10,12,21^ A common challenge is the development and selection of suitable reversible binder molecules. Most works have focused on measuring small-molecule targets and the use of aptamers as reversible binder molecules. However, compared to aptamers, antibodies have higher diversity in their physicochemical properties such as charge and hydrophobicity, which is advantageous for achieving high binding specificity, particularly in case of protein biomarkers.^37,38^

In this research, we have demonstrated the feasibility of using antibody fragments to achieve continuous protein biosensing with spontaneous sensor reversibility. Custom-designed Fab fragments with rapid association and dissociation kinetics were implemented in a competition-based biosensor, enabling continuous lactoferrin detection at nanomolar concentrations using a sensor format with a single antibody fragment, without reagent consumption or a regeneration method. Future research will focus on enhancing sensitivity into the picomolar range using a sandwich format, developing sensors for other protein biomarkers, and on further studying sensor kinetics and analytical performance including imprecision and accuracy, all studied with varied complex matrices. We envision that reversible antibody fragments can be applied in several biosensing platforms for achieving continuous protein biosensing, in order to study and control time-dependent processes in fields such as fundamental biological research, patient monitoring, and industrial bioprocessing.

## Materials and Methods

### Materials

PBS tablets, NaCl and bovine serum albumin (BSA) were ordered at Sigma-Aldrich. Bovine lactoferrin was supplied by FrieslandCampina. Thirteen custom HuCAL recombinant antibodies (Fabs) against bovine lactoferrin were produced by Bio-Rad.^17,20^ The oligonucleotides used in this study were purchased from IDT. Polystyrene slides (25 × 75 mm) were laser cut from polystyrene sheets (transparent) obtained from Goodfellow. Custom-made flow cell stickers with a surface area of 44 mm^2^ and height of 450 μm were purchased from Grace Biolabs (USA). Custom-made cyclic olefin copolymer (COC) cartridges containing a flow chamber (20 μL volume, 250 μm chamber height), compatible with the custom-built automated setup, were produced by Axxicon using injection molding.

### Fab Biotinylation

The SpyTag/SpyCatcher technology was used for site-directed conjugation of biotin to the recombinant Fabs.^39^ SpyTag2-Fab and SpyCatcher2-biotin (TZC001B; Bio-Rad) were diluted in PBS and incubated for 2 h at RT at a final concentration of 6 and 5 μM (1.2:1), respectively. The biotinylated Fabs were aliquoted and stored in the freezer (−20 °C). The SpyTag-SpyCatcher conjugation was confirmed using SDS-PAGE (Supporting Information Figure S3).

### f-BPM Screening

#### f-BPM Particle Functionalization

2 μL streptavidin-coated Dynabeads MyOne C1 (10 mg/mL; Thermo Fisher Scientific) were incubated with 2 μL 50 nM biotin-Fab for 30 min at room temperature (RT) on a rotating fin (VWR, The Netherlands). Subsequently, 100 μL of 100 μM biotin-mPEG (1 kDa; Nanocs) in PBS was added and incubated for 30 min at RT on the rotating fin. The particle mixtures were put against a magnet to collect the particles, the solution was removed and 50 μL 0.2 mg/mL poly(l-lysine)-grafted-poly(ethylene glycol) (PLL(20)-g[3.5]-PEG(2); SuSoS) was added to block the particle surface. After 2 h of incubation at RT on the rotating fin, the particles were washed with 1 mL PBST (PBS with 0.05% Tween-20) using a magnet rack. The particles were resuspended in PBS with 500 mM NaCl (HS buffer) and sonicated for 10 s in a sonication bath. Finally, the particles were diluted 20 times in HS buffer.

#### 96-Well Plate Preparation

The polyclonal anti-lactoferrin antibody (A10-126A; Thermo Fisher Scientific) was diluted to 50 nM in carbonate coating buffer (0.05 M carbonate, pH 10) and 50 μL was added to each well of a transparent 96-well plate (Nunc MaxiSorb flat-bottom; Thermo Fisher Scientific). The plate was sealed and incubated for 1 h at RT. Next, the coating solution was removed and 100 μL blocking buffer (PBS with 1% BSA) was added and incubated for 1 h at RT. Subsequently, the blocking solution was removed and 40 μL of the diluted particles were added. Lastly, 10 μL lactoferrin (1 pM–1 nM) was added and the samples were incubated for 1 h before measuring.

#### Flow Cell Preparation

The custom-made flow cell sticker was mounted on a polystyrene slide. One slide can contain a maximum of 12 flow cells. All additions were done manually using a micropipet and a volume of 50 μL (flow cell volume is ∼20 μL). The polyclonal anti-lactoferrin antibody was diluted to 50 nM in carbonate coating buffer and added to each flow cell. The residual liquid at the outlet was removed and the flow cells were incubated for 1 h at RT in a humidity chamber to prevent evaporation. Subsequently, the blocking buffer was added to each flow cell and incubated for 1 h at RT in a humidity chamber. The blocking buffer was washed away using HS buffer. Next, the diluted particles were added and the flow cells were incubated for 30 min to allow the particles to sediment to the surface. Next, 500 pM lactoferrin, diluted in HS buffer, was added and the flow cells were immediately measured repeatedly over time. The inlets and outlets of the flow cells were sealed to prevent evaporation of the fluid.

#### Screening Measurements

The f-BPM screening measurements (96-well plate and flow cells) were performed using a custom-built bright-field microscope containing a motorized *XY* stage (ASR series; 100 mm × 120 mm travel; Zaber). A 10 times magnification (10× DIN achromatic finite intl standard objective; Edmund Optics), simple 3 mm green led (12 V) and 3.2 MP camera (Flir BFS-U3–32S4M-C) with a field of view of 0.71 mm × 0.53 mm (pixel size 345 nm) were used to visualize the particles. A miniature linear actuator (Zaber T-LA13A) was used for the autofocus. The custom-built microscope was controlled using MATLAB. The positions to be measured were set (different flow cells on one slide or different wells of a 96-well plate) and each position was measured for 0.2 min at a framerate of 30 Hz. Multiple series of all positions were measured, allowing to measure the response over time. The frames of each measurement were analyzed in real-time using particle tracking software described by Bergkamp et al.^16^ The diffusivity time traces were obtained from the particle tracking data. The bound fraction is the output parameter that is derived from the diffusivity time traces (Supporting Information Figure S1).

#### Binding Analysis Using SPR

Biacore X100 (Cytiva) and a DNA chip (RGD200M; XanTec bioanalytics GmbH) were used for the binding analysis of the 13 Fabs. DNA streptavidin (RG-SA; XanTec bioanalytics GmbH) was diluted 1:100 in the running buffer (PBS with 500 mM NaCl and 0.005% P20) and captured in channel 1 and 2 for 5 min at a flow rate of 5 μL/min. Subsequently, 2 nM Biotin-Fab (diluted in running buffer) was captured only in channel 2 for 5 min at 5 μL/min, channel 1 was used as the reference. 500 nM lactoferrin (diluted in running buffer) was flown for 180 s at a 30 μL/min flow rate in both channels. Dissociation was measured for 15 min, flowing the running buffer at 30 μL/min. Both channels were regenerated using 0.05 M NaOH with 1 M NaCl for 60 s at 10 μL/min.

### Continuous t-BPM Sensor

#### Preparation of ssDNA-Lactoferrin Conjugate

100 μL of lactoferrin protein (10 mg/mL) in PBS was mixed with 3.13 μL 20 mM TFP Ester-PEG4-DBCO (C20039, Thermo Fisher Scientific) in DMSO and incubated at RT for 30 min. Then the DBCO-modified lactoferrin was purified with a zeba-desalting column (89882; Thermo Fisher Scientific) according to the manufacturer’s instructions. Afterward, 50 μL DBCO-lactoferrin in PBS (10 mg/mL) was mixed with 50 μL of 500 μM azide-modified oligonucleotide (Supporting Information Table S1) at 4 °C overnight. The ssDNA-lactoferrin conjugate was then reconstituted with BSA at a final concentration of 0.01% and stored at 4 °C.

#### t-BPM Particle Functionalization

2 μL streptavidin-coated Dynabeads MyOne C1 (10 mg/mL; Thermo Fisher Scientific) were incubated with 4 μL 50 nM biotin-Fab for 30 min at RT on a rotating fin. The remaining biotin binding sites were partly blocked by adding 1.25 μL 10 μM biotin-mPEG (1 kDa, Nanocs) and 5 μL PBS. After 40 min of incubation at RT on the rotating fin, the particles were washed with 1 mL PBST, resuspended in 100 μL HS buffer and sonicated for 10 s.

#### Cartridge Preparation

A polymer mixture of 0.45 mg/mL poly(l-lysine)-grafted-poly(ethylene glycol) (PLL(20)-g[3.5]-PEG(2); SuSoS) and 0.05 mg/mL azide functionalized PLL-*g*-PEG (PLL(15)-g[5]-PEG(2)-N3; Nanosoft Biotechnology LCC) was prepared in ultrapure MQ, as described by Lin et al.^15^ The COC cartridges were rinsed twice with Milli-Q and cleaned using 15 min of sonication in Milli-Q. The cartridges were dried using a nitrogen steam and exposed to UV Ozone (UV Ozone Cleaner, Novascan) for 30 min. Immediately after the UV Ozone treatment, the flow chamber of the cartridge was sealed with an adhesive film (Optically Clear Adhesive Seal Sheets; Thermo Fisher Scientific) and the PLL-*g*-PEG/PLL-*g*-PEG-N3 mixture was injected. After 3 h of incubation at RT in a humidity chamber, the free polymer was removed by extracting the solution from the chamber. Subsequently, a DNA mixture was added, containing 0.4 nM dsDNA tether (DBCO modified) and 3 μM DBCO-ssDNA (docking DNA) diluted in HS buffer (sequences are shown in Supporting Information Table S1). The inlet and outlets of the flow chamber were sealed and the cartridges were stored for at least 3 weeks, up to 3 months.

#### Cartridge Activation

A prepared cartridge was washed with HS buffer and freshly prepared particles were added manually using a pipet. The cartridge was inserted into the custom-built automated setup and the particles were incubated for 10 min to allow the particles to become bound to the biotin moiety on the substrate-side dsDNA tether. From this point on, all additions were done automated with a syringe pump at a flow rate of 100 μL/min and a flushing volume of 100 μL. After particle tethering, the remaining biotin-binding sites were blocked with 100 μM 1 kDa biotin-mPEG for 20 min. The system was activated using 1 nM LF-ssDNA, which hybridizes to the substrate-side docking DNA. The bound fraction was continuously measured, to monitor the activation process. When a bound fraction of approximately 0.6 was obtained, the activation process was stopped by flushing HS buffer and the baseline level was measured. The cartridge was then ready to measure lactoferrin samples. All samples were flushed through the flow chamber with a flow rate of 100 μL/min for 1 min and the measurement time was varied depending on the type of measurement.

#### Automated Setup and Data Analysis

A Laboratory Programmable Syringe Pump (LSPone) and 12-port rotary valve from AMF were used to transport different samples. PTFE tubing (BL-PTFE-1608–20) and connectors (CIL-XP-245X) from Darwin Microfluidics were used to connect the LSPone pump and valve to the cartridge. A cartridge holder and a sample holder were custom-made from aluminum. O-rings were used to ensure the connections with the connectors in the cartridge holder and the holes in the cartridge were watertight. The particles inside the flow chamber were tracked using a custom-made optical setup containing a 10 times magnification (10× DIN achromatic finite intl standard objective (Edmund Optics)), 3 mm green led (12 V) and 3.2 MP camera (Flir BFS-U3-32S4M-C) with a field of view of 0.71 mm × 0.53 mm (pixel size 345 nm). A miniature linear actuator (Zaber T-LA13A) was used for the autofocus. The complete setup (pumps, valves and microscope) was controlled using MATLAB. Continuous measurements of 1 min at a framerate of 30 Hz were performed after a sample was flushed through the flow chamber, so in the absence of flow. The frames of each measurement were analyzed in real-time using particle tracking software described by Bergkamp et al.^16^

## Supporting Information Available

BPM sensing principle, more details on the sensor components, dose–response curves over time, 18 h kinetic measurement, fitted SPR data, original data of different Fabs tested in the t-BPM sensor, signal drift correction, and time dependencies in the t-BPM sensor response (PDF)
