# Supplementary material for: Continuous Protein Sensing Using Fast-Dissociating Antibody Fragments in Competition-Based Biosensing by Particle Motion
Source: ACS Sens. 2025 Mar 24;10(4):2895–905. doi: 10.1021/acssensors.4c03637 (PMC12038830; doi:10.1021/acssensors.4c03637)
Supplement: Supplementary file 1 — se4c03637_si_001.pdf [file se4c03637_si_001.pdf]

Supporting Information

Continuous protein sensing using fast-dissociating antibody fragments in competition-based biosensing by particle motion

Claire M.S. Michielsen[1,3], Yu-Ting Lin[4], Junhong Yan[4], Arthur M. de Jong[2,3], Menno W.J. Prins[1,2,3,4,*]

[1] Department of Biomedical Engineering, Eindhoven University of Technology, Eindhoven 5612 AE, The Netherlands

[2] Department of Applied Physics, Eindhoven University of Technology, Eindhoven 5612 AE, The Netherlands

[3] Institute for Complex Molecular Systems (ICMS), Eindhoven University of Technology, Eindhoven 5612 AE, The Netherlands

[4] Helia Biomonitoring, Eindhoven 5612 AR, The Netherlands

*Corresponding author. Email: m.w.j.prins@tue.nl

Table of Contents

1. List of supporting information Figures and Tables S2
2. Biosensing by Particle Motion sensing principle S3
3. Sensor components........ S5
   3.1 DNA sequences S5
   3.2 Lactoferrin DNA conjugation S6
   3.3 Biotin conjugation to Fab via SpyTag/SpyCatcher system........ S7
4. Screening of Fabs using Biosensing by free Particle Motion (f-BPM) S8
   4.1 Dose-response curves over time........ S8
   4.2 Kinetic measurement in flow cells S10
5. Mapping binder kinetics using Surface Plasmon Resonance........ S11
   5.1 Association and dissociation data of all Fabs S11
   5.2 Single-cycle SPR data for Fabs with rapid association and dissociation S12
6. Continuous lactoferrin sensing using t-BPM S15
   6.1 Raw data Fab 5, 6, 7, 8, 12 and 13 in t-BPM........ S15
   6.2 Signal drift correction S16
   6.3 Sensor response in buffer and milk samples........ S17
   6.4 Time dependencies in the t-BPM sensor response S18
References........ S20

1. List of supporting information Figures and Tables

Supporting Information Figures

Figure S1. Biosensing by Particle Motion sensing principle……………………………………………S4

Figure S2. SDS-PAGE gel of ssDNA conjugation to lactoferrin……………………………………..S6

Figure S3. Conjugation of SpyTag-Fab with Biotin-SpyCatcher…………………………………….S7

Figure S4. f-BPM signal responses to varying lactoferrin concentrations measured in a 96-well plate (Fabs 3, 4, 6, 7, 8, 12, and 13)….………………………………………………………………………S8

Figure S5. f-BPM signal responses to varying lactoferrin concentrations measured in a 96-well plate (Fabs 1, 2, 4, 9, 10, and 11)…………………………….………………………………..…..S9

Figure S6. Time-dependent f-BPM sensor signals for each Fab following lactoferrin addition......S10

Figure S7. Association and dissociation SPR data fitted with a single exponential equation……..S11

Figure S8. Single-cycle kinetic measurements for Fabs with rapid association and dissociation..S13

Figure S9. t-BPM sensor response over time at varying lactoferrin concentrations……………….S15

Figure S10. Signal drift correction using linear interpolation between the highest and lowest signal points………………………………………………………………………………………………..S16

Figure S11. Lactoferrin measurements in buffer and milk samples using the competition-based t-BPM sensor…..…………………………………………………………………………..….S17

Figure S12. Analysis of time-dependency in the t-BPM sensor response..………………………...S18

Figure S13. Effect of repeated flushing on t-BPM sensor response..………………………………S19

Supporting Information Tables

Table S1. DNA sequences used in the t-BPM sensor…………………………………………………..S5

Table S2. Final signal intensities and response times for Fabs from time-dependent f-BPM measurements……………………………………………………………………………..S10

Table S3. Association and dissociation rate constants for Fabs from SPR data……………………S11

Table S4. Dissociation constants extracted from single exponential fits in the inset of Figure S8A…………………………………………………………………………………………….….S14

Table S5. Association parameters extracted from single exponential fits in Figure S8…………….S14

Table S6. Equations of the linear fit between background and baseline data points for each series……………………………………………………………………………………………S16

Table S7. Signal rate parameters of the t-BPM sensor response after analyte addition…..……....S19

Table S8. Signal rate parameters of the t-BPM sensor after blank addition………………………...S19

2. Biosensing by Particle Motion sensing principle

Biosensing by Particle Motion (BPM) relies on reversible interactions between biofunctionalized particles and a biofunctionalized sensor surface. The measurements are based on tracking the motion of these particles. When the particles are not interacting with the surface, they move freely, exhibiting high diffusion coefficients. Conversely, interactions with the surface limit the particle motion, resulting in lower diffusion coefficients. By utilizing brightfield video microscopy and particle tracking software, hundreds to thousands of particles are analyzed simultaneously[1]. In this study, the bound fraction is used as the sensor output, representing the ratio of bound states to the total observed states within the measurement time. Other read-out parameters of the BPM technology are explained by Buskermolen *et al.*[2]

BPM operates in two formats: tethered BPM (t-BPM) and free BPM (f-BPM). In t-BPM, particles are tethered to the sensor surface using double-stranded DNA (Figure S1A), keeping them in close proximity to the surface and ensuring a high encounter rate[3,4]. The tether also prevents particle drift in case of fluid flow. Different tether lengths have been tested in BPM, with the goal of maintaining the particle's proximity to the surface while preserving enough contrast between bound and unbound states[5]. In this study, a 75 nm tether was used, which strikes a balance between proximity and state differentiation. A t-BPM sensor requires preparation steps that incorporate a tether between particle and surface. f-BPM utilizes free particles, without a tether between particle and surface, which simplifies sensor preparation and is suited for high-throughput screening (Figure S1B)[2]. However, free particles can drift and be lost during washing steps, making t-BPM more suitable for analysis of multiple samples with a single sensor.

In this study, particles with a 1 μm diameter were used, in both t-BPM and f-BPM. While other particle sizes can be employed, they come with trade-offs: smaller particles experience less drag and move faster, whereas larger particles move more slowly and remain closer to the sensor surface due to their larger mass, leading to a higher encounter rate[2]. The choice of 1 μm particles was taken from our earlier works.

BPM can be applied in both competition and sandwich formats. In a competition format (Figure S1A), one analyte-specific binder is used and one analyte-analogue. Without analyte, particles bind frequently to the sensor surface, shifting the distribution to low diffusion coefficients (Figure S1C left). Analyte presence reduces interactions with the sensor surface due to competitive binding, shifting the distribution to higher diffusion coefficients (Figure S1C right). In a sandwich format (Figure S1B), particles and sensor surfaces are functionalized with binders that can both bind to the analyte molecule simultaneously. Without analyte, particles move freely, resulting in a distribution with high diffusion coefficients (Figure S1D, left). Analyte presence promotes sandwich binding between the particles, analyte, and the sensor surface, restricting particle motion and shifting the distribution to lower diffusion coefficients (Figure S1D right).

In both assay formats, multivalent interactions can occur when multiple binding sites on a particle interact simultaneously with the sensor surface, which stabilizes the bound state of the particle. Multivalent interactions can be caused by heterogeneous binder immobilizations[6]. Optimizing binder densities is essential to limit multivalent binding and achieve a reversible sensor. Analyzing bound-state lifetimes and diffusion coefficients can provide insights into the occurrence of multivalent binding in a sensor.

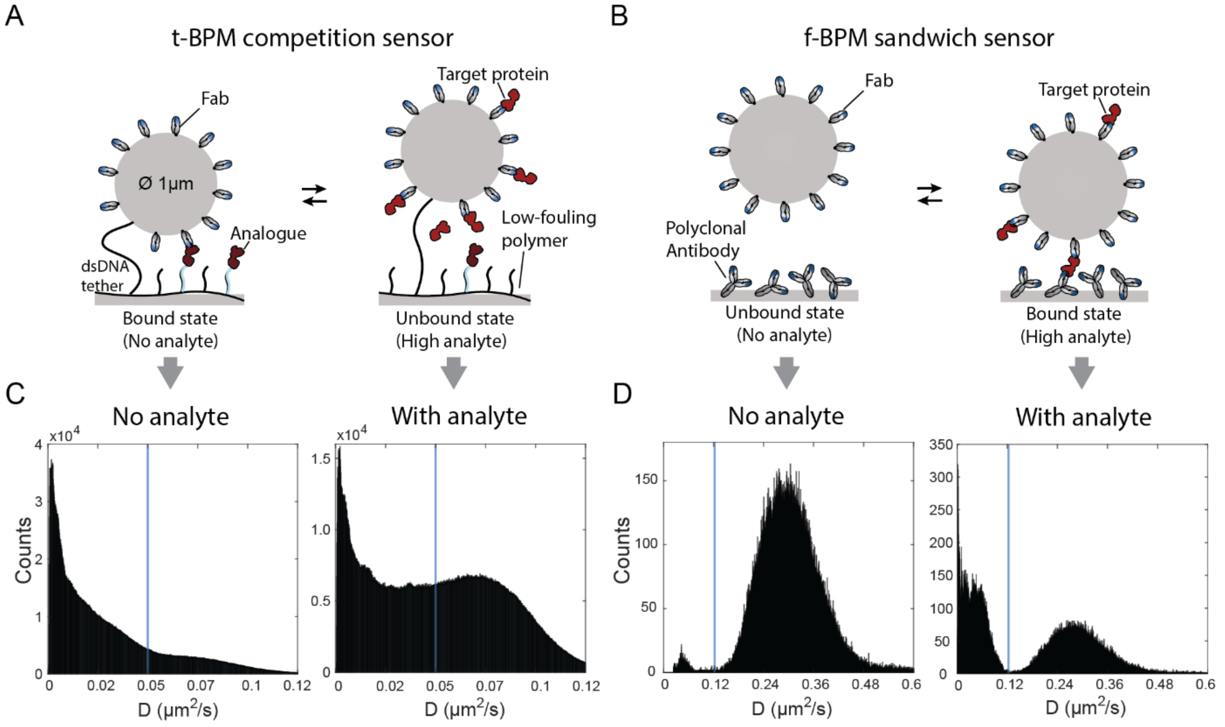

Figure S1. Biosensing by Particle Motion (BPM) sensing principle. *(A) Schematic of a competition-based tethered BPM (t-BPM) sensor. Particles are coated with molecular binders (Fabs) and tethered to a low-fouling polymer on the sensor surface. Analogue molecules are also immobilized on the low-fouling polymer. In the absence of the target protein (analyte), particles bind frequently to the analogue molecules on the surface. In the presence of the target analyte, particle interactions with the analogue molecules are reduced due to competitive binding. (B) Schematic of a sandwich-based free BPM (f-BPM) sensor. Particles are coated with molecular binders (Fabs) and the sensor surface is coated with polyclonal antibodies. In the absence of the target analyte, particles move freely over the sensor surface. When the analyte is present, sandwich binding between the particles, analyte, and the sensor surface restricts particle motion. (C) Diffusion coefficient (D) histograms of all particles in the competition-based t-BPM sensor. Without analyte (left), the distribution exhibits low diffusion coefficients (D<0.05 μm^2/s), indicating bound states. In the presence of analyte (right), the proportion of unbound states increases, reflected by higher diffusion coefficients (D>0.05 μm^2/s). (D) Diffusion coefficient histograms of all particles in the sandwich-based f-BPM sensor. Without analyte (left), particles are mostly in the unbound state, resulting in diffusion coefficients above the threshold (0.12 μm^2/s). In the presence of analyte (right), the freely diffusing population (D>0.12 μm^2/s) has decreased and the bound population has increased (D<0.12 μm^2/s) due to sandwich bonds between particle binders and sensor surface binders. The blue lines in panel C and D represent the threshold used to determine the bound fraction.*

3. Sensor components

This section describes the biomolecular components and conjugation strategies utilized in the study. It starts with an overview of the DNA sequences used in the sensor, followed by the conjugation of DNA to lactoferrin, and finally, the biotinylation of Fabs using the SpyTag/SpyCatcher system.

3.1 DNA sequences

Three types of DNA sequences were used in the sensor, as detailed in Table S1. Double-stranded DNA (dsDNA) with biotin at one end and DBCO at the other was employed to tether the particles to the sensor surface. The DBCO group facilitated conjugation of the dsDNA to the sensor surface, which was coated with a low-fouling polymer mixture of poly(L-lysine)-grafted-poly(ethylene glycol) (PLL-g-PEG) and PLL-g-PEG-azide. The biotin moiety enabled the capture of streptavidin-coated particles. The dsDNA with these functional groups was prepared as described by Yan et al., 2020[7].

In addition to the dsDNA tether, the low-fouling polymer on the sensor surface was functionalized with DBCO-functionalized single-stranded DNA (DBCO-ssDNA). These ssDNA molecules were used to immobilize ssDNA-functionalized lactoferrin molecules via DNA hybridization. Lactoferrin was functionalized with complementary ssDNA through a two-step process: first, DBCO was conjugated via EDC/NHS coupling; then, azide-ssDNA was attached via click chemistry, further explained in section 3.2.

Table S1. DNA sequences used in the t-BPM sensor.

	**Sequence**
**dsDNA tether**	5′ DBCO-GGT TAG CAG CCT GTT TCA AAA CCT GGG GGT GAG TGT CAC GCC AAT TCA GCG CAT CGT TCT GTC GGG AGA GAA TGG TCT GAA AAT CGA TAT CCA CGT CAT TAT CCC GTA CGA AGG TCT TTC TGG TGA TCA GAT GGG GCA GAT AGA AAA AAT ATT CAA AGT GGT GTA CCC AGT AGA CGA TCA TCA CTT CAA GGT TAT ACT GCA CTA TGG CAC CCT CGT TAT CG 3′<br><br>3′ CCA ATC GTC GGA CAA AGT TTT GGA CCC CCA CTC ACA GTG CGG TTA AGT CGC GTA GCA AGA CAG CCC TCT CTT ACC AGA CTT TTA GCT ATA GGT GCA GTA ATA GGG CAT GCT TCC AGA AAG ACC ACT AGT CTA CCC CGT CTA TCT TTT TTA TAA GTT TCA CCA CAT GGG TCA TCT GCT AGT AGT GAA GTT CCA ATA TGA CGT GAT ACC GTG GGA GCA ATA GC-biotin 5′
**DBCO-ssDNA**	5′ DBCO-GTG CGG CAG GGG TAA GAC CA 3’
**LF-ssDNA**	5' Azide-TTT TTT TTG GTC TTA CCC CTG CCG CAC-3'

3.2 Lactoferrin DNA conjugation

Lactoferrin was immobilized on the sensor surface to facilitate competitive binding with free lactoferrin molecules. In the absence of free lactoferrin, particles bind to the sensor surface, whereas in the presence of lactoferrin, the binding of particles is reduced. Immobilization was achieved via DNA hybridization between ssDNA attached to lactoferrin and complementary ssDNA anchored to the low-fouling polymer-coated surface.

The attachment of ssDNA to lactoferrin was accomplished through a two-step conjugation process. First, EZ-Link™ TFP Ester-PEG4-DBCO was conjugated to the primary amines on lactoferrin via EDC/NHS coupling. In the second step, azide-ssDNA (Table S1) was conjugated to the DBCO moiety on lactoferrin through click chemistry. The protocol of the conjugation can be found in the Materials and Methods section.

The success of the conjugation was analyzed using SDS-PAGE, as shown in Figure S2. Protein staining reveals a prominent dark band just above 75 kDa, consistent with the molecular weight of lactoferrin (80 kDa). In the sample containing lactoferrin conjugated to DNA, additional bands at higher molecular weights are observed, indicating successful conjugation. These bands suggest that some lactoferrin molecules are conjugated to one, two, or more DNA strands.

DNA staining also shows the presence of DNA in the bands above 80 kDa. However, a band corresponding to unmodified lactoferrin is also detected in the DNA-stained gel, indicating some non-specific interaction of the DNA stain with lactoferrin. Comparing the protein staining and DNA staining intensities leads to the conclusion that the intense bands in the LF-DNA sample result from DNA conjugation to lactoferrin.

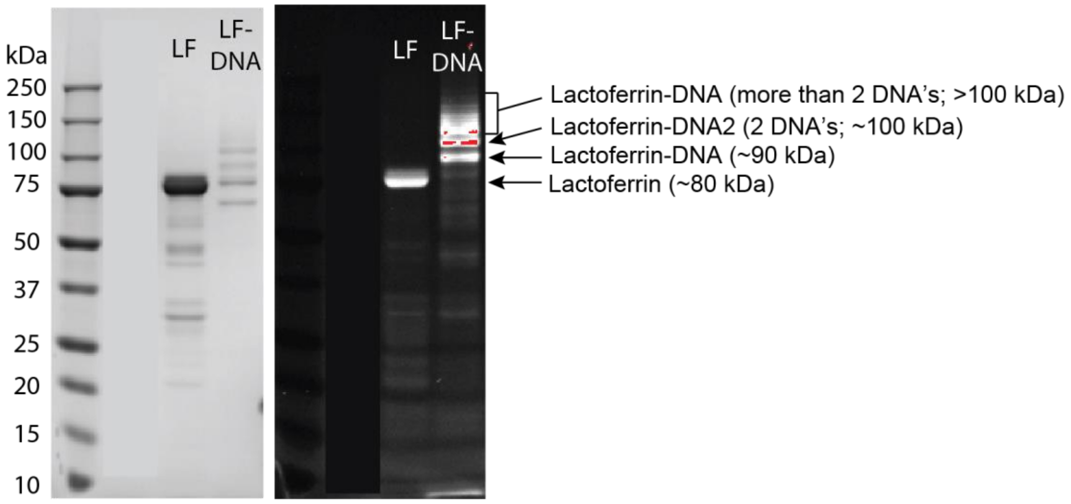

Figure S2. SDS-PAGE gel of ssDNA conjugation to lactoferrin. *Protein staining (dark bands) is shown on the left, and DNA staining (white bands) is shown on the right. Red regions indicate saturated pixels.*

This method of DNA conjugation via primary amines, commonly found in proteins due to lysine residues, is generalizable to other proteins with similar amine groups. However, limitations may arise when lysines are located within a binding site or if the protein is very small, as conjugation could affect binder accessibility. Alternative more site-specific conjugation strategies, such as site-specific conjugation via a cysteine (maleimide-thiol conjugation), could be explored to improve or tailor conjugation efficiency, depending on the target protein's properties.

3.3 Biotin conjugation to Fab via SpyTag/SpyCatcher system

Custom-made recombinant Fabs, provided by Bio-Rad, were equipped with a SpyTag, a 13-amino-acid peptide that forms a stable covalent isopeptide bond with its protein partner, SpyCatcher (15.6 kDa)[8]. Biotin-SpyCatcher, also obtained from Bio-Rad, was used for site-specific biotinylation of the Fabs (Figure S3A). The detailed conjugation protocol is described in the Materials and Methods section.

The conjugation was evaluated using SDS-PAGE. Figure S3B illustrates the analysis of the unconjugated proteins. The recombinant Fabs feature a SpyTag2 at the C-terminus of the heavy chain, and there is no disulfide bond between the heavy and light chains. Therefore, two bands are observed on the gel: the upper band (near the 25 kDa marker) represents the heavy chain with the SpyTag, and the lower band (just above the 20 kDa marker) corresponds to the light chain. Biotin-SpyCatcher appears as a band just above the 15 kDa marker, consistent with its molecular weight of 15.6 kDa.

Figure S3C displays the conjugation products for all Fabs (1–13). The absence of a band for biotin-SpyCatcher indicates that all SpyCatchers were conjugated to the SpyTags, forming a product that appears just below the 50 kDa marker (biotin-SpyCatcher-SpyTag-HeavyChain). The band just above the 20 kDa marker corresponds to the light chain of the Fab. Additionally, a band just above the 25 kDa marker represents the SpyTag-Fab heavy chain. This band persists due to the excess of Fab used during the conjugation.

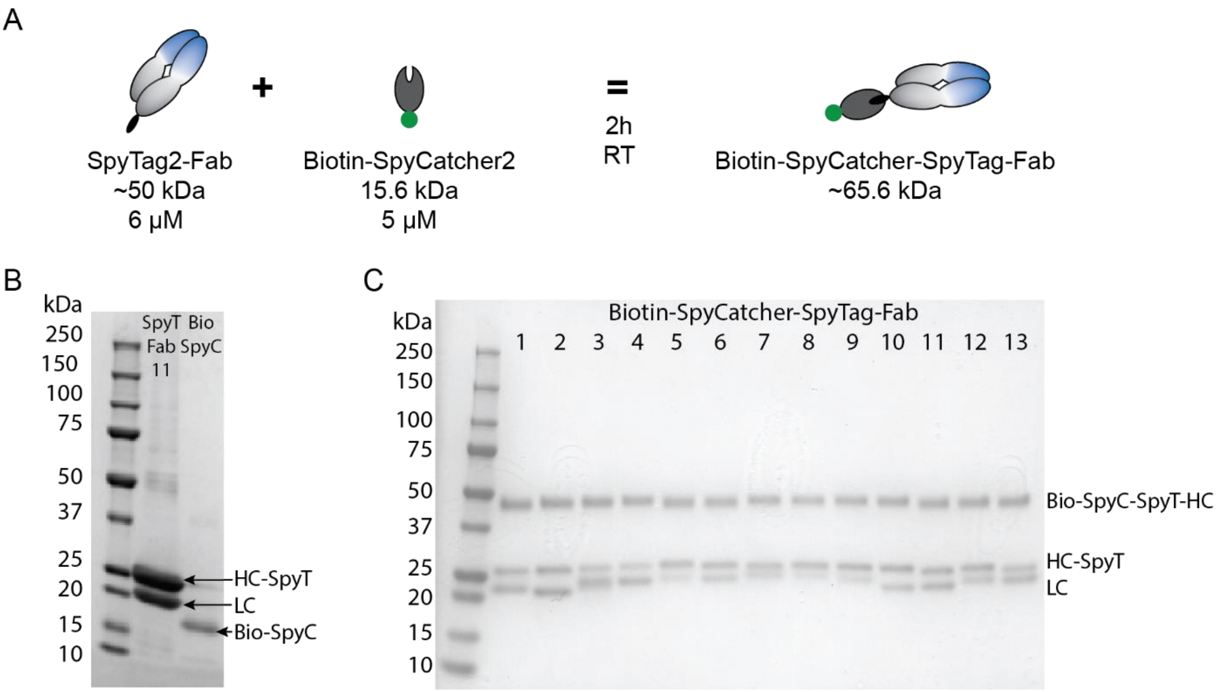

Figure S3. Conjugation of SpyTag-Fab with Biotin-SpyCatcher. *(A) The Fabs contain a SpyTag2 at the C-terminus of the heavy chain and Biotin-SpyCatcher2 contains a single biotin molecule. The Fab and SpyCatcher were incubated at a 1.2:1 ratio for 2 hours at room temperature. (B) SDS-PAGE gel of the unconjugated components: SpyTag2-Fab (left) next to the protein standard and Biotin-SpyCatcher (right). (C) SDS-PAGE gel of the conjugation products of all Fabs (1-13), with the protein standard visible on the left.*

4. Screening of Fabs using Biosensing by free Particle Motion (f-BPM)

4.1 Dose-response curves over time

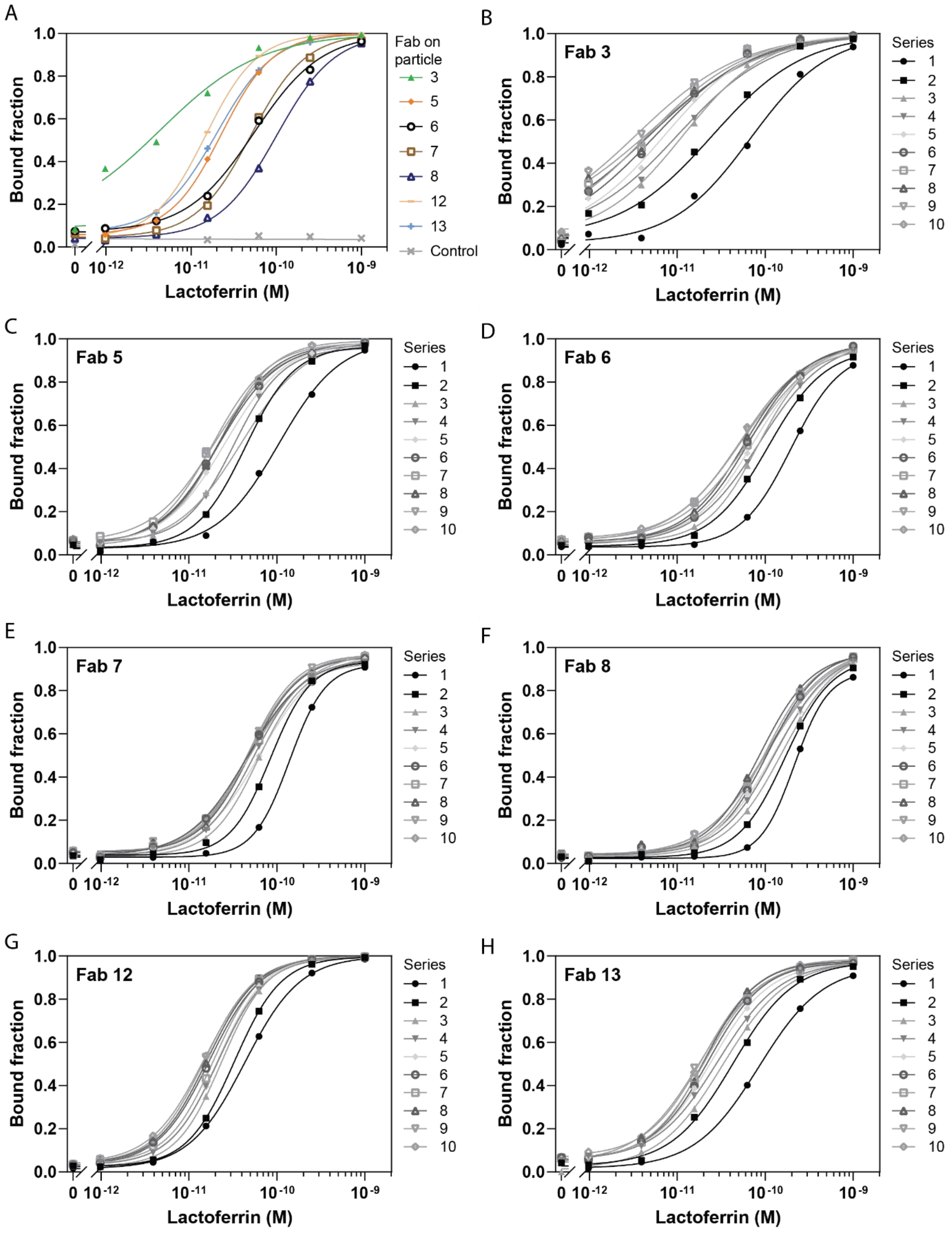

Figure S4. f-BPM signal responses to varying lactoferrin concentrations measured in a sandwich assay in a 96-well plate. *(A) Dose-response curves for Fabs 3, 4, 6, 7, 8, 12, and 13, represented by distinct colors and symbols, measured using polyclonal anti-lactoferrin antibodies on the substrate and Fabs immobilized on particles. Particles without immobilized Fabs served as negative control (grey crosses). The data corresponds to measurement series 10. (B–H) Dose-response curves for individual Fabs: Fab 3 (B), Fab 5 (C), Fab 6 (D), Fab 7 (E), Fab 8 (F), Fab 12 (G), and Fab 13 (H). Each measurement series is distinguished by unique symbols and varying shades of grey. The time interval between consecutive series is approximately one hour.*

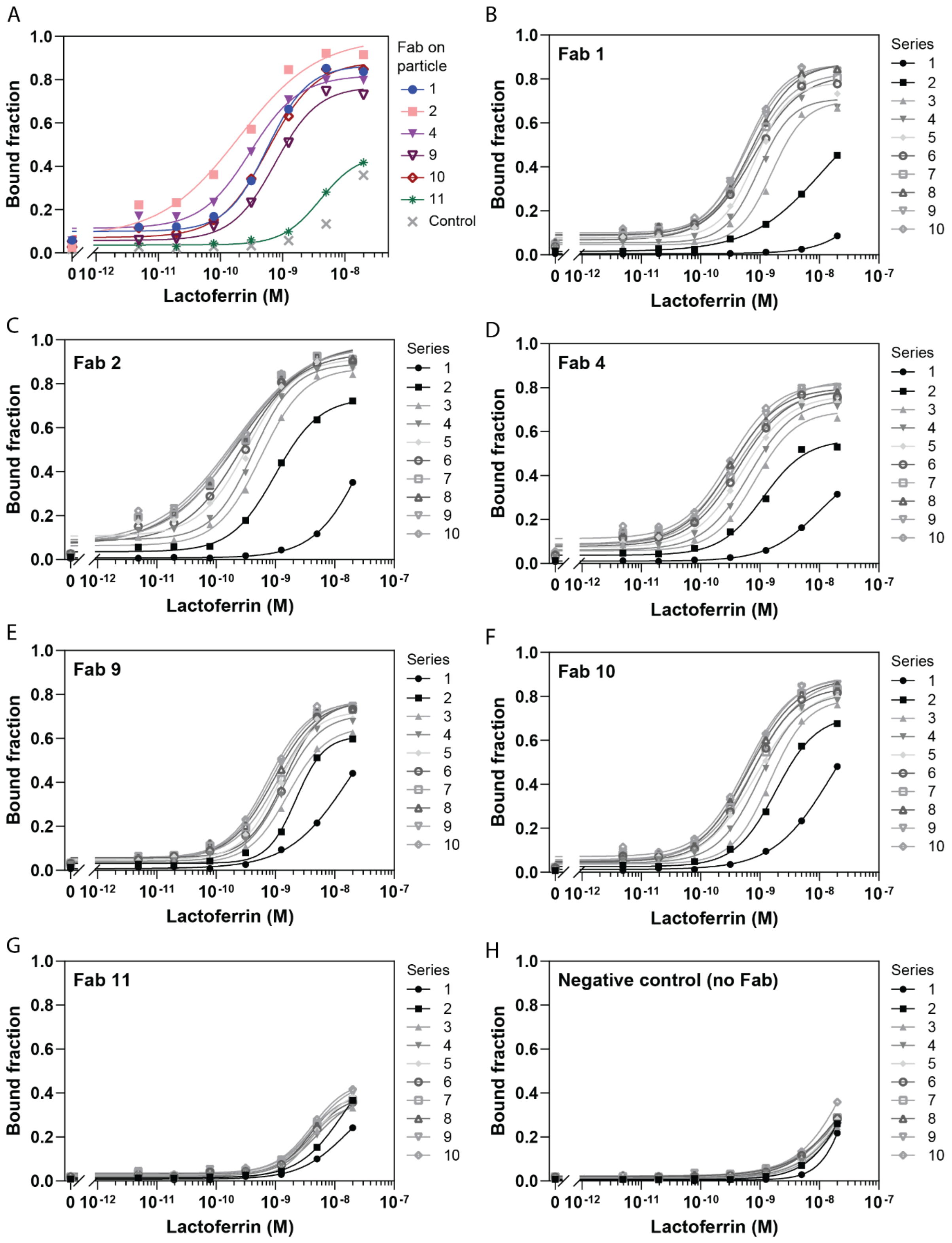

Figure S5. f-BPM signal responses to varying lactoferrin concentrations measured in a sandwich assay in a 96-well plate. *(A) Dose-response curves for Fabs 1, 2, 4, 9, 10, and 11, represented by distinct colors and symbols, measured using polyclonal anti-lactoferrin antibodies on the substrate and Fabs immobilized on particles in a 96-well plate. Particles without immobilized Fabs served as negative control (grey crosses). The data corresponds to measurement series 10. (B–H) Dose-response curves for individual Fabs: Fab 1 (B), Fab 2 (C), Fab 3 (D), Fab 9 (E), Fab 10 (F), Fab 11 (G), and the negative control (H). Each measurement series is distinguished by unique symbols and varying shades of grey. The time interval between consecutive series is approximately one hour.*

4.2 Kinetic measurement in flow cells

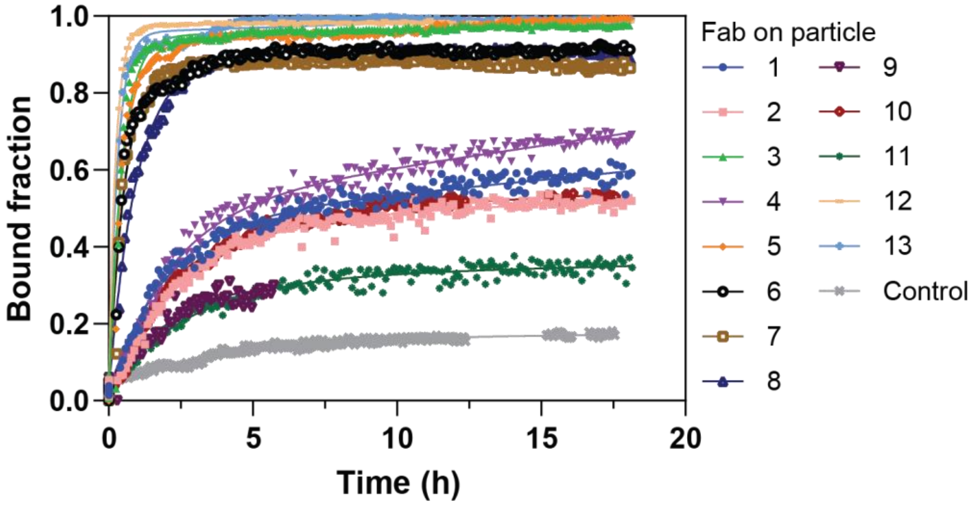

Figure S6. Time-dependent f-BPM sensor signals for each Fab following lactoferrin addition. *Time-dependent signals were measured in a static flow cell after the addition of 500 pM lactoferrin. Data points are shown as symbols, while solid lines represent the fits using Equation 1. Measurements were conducted over 18 hours for all Fabs.*

Table S2. Final signal intensities and response times for Fabs from time-dependent f-BPM measurements. *Final signal values and characteristic response times were extracted from the fit of time-dependent curves in Figure S6 using Equation 1.*

**Fab**	**S_{end}**		**Characteristic response rate (h^{-1})**		**Characteristic response time (h)**	**Goodness of fit**
	Value	95% CI	Value	95% CI	$\tau = 1/k$	R^2
**1**	0.46	0.45 to 0.48	0.507	0.466 to 0.551	1.97	0.984
**2**	0.47	0.45 to 0.48	0.392	0.363 to 0.421	2.55	0.986
**3**	0.95	0.94 to 0.96	2.172	2.030 to 2.327	0.46	0.978
**4**	0.50	0.48 to 0.51	0.479	0.442 to 0.519	2.09	0.986
**5**	0.92	0.91 to 0.93	2.149	2.055 to 2.248	0.47	0.993
**6**	0.88	0.87 to 0.88	1.785	1.693 to 1.884	0.56	0.989
**7**	0.89	0.88 to 0.89	1.762	1.680 to 1.849	0.57	0.989
**8**	0.89	0.89 to 0.90	1.036	1.010 to 1.064	0.97	0.997
**9**	3.60	0.42 to -	0.090	-0.161 to 0.371	11.10	0.959
**10**	0.48	0.47 to 0.48	0.418	0.4021 to 0.435	2.39	0.993
**11**	0.32	0.30 to 0.34	0.360	0.317 to 0.406	2.78	0.969
**12**	0.98	0.98 to 0.98	4.192	4.119 to 4.268	0.24	1.000
**13**	0.96	0.95 to 0.96	3.303	3.116 to 3.512	0.30	0.993

5. Mapping binder kinetics using Surface Plasmon Resonance

5.1 Association and dissociation data of all Fabs

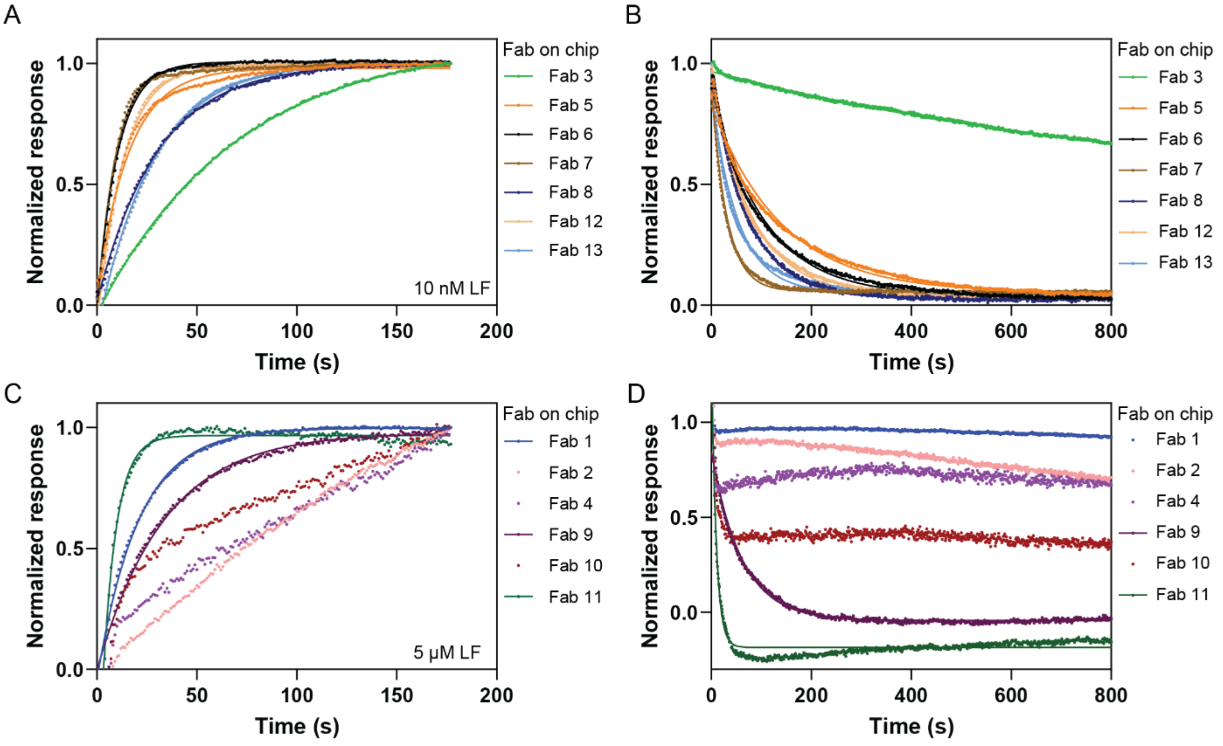

Figure S7. Association and dissociation SPR data fitted with a single exponential equation. *(A) Association curves (10 nM Lactoferrin) and (B) dissociation curves for Fab 3, 5, 6, 7, 8, 12 and 13 immobilized on the sensor chip. (C) Association curves (5 μM Lactoferrin) and (D) dissociation curves of Fab 1, 2, 2, 9, 10 and 11 immobilized on the sensor chip. Due to signal instability caused by nonspecific interactions at high lactoferrin concentrations, data for Fabs 2, 4, and 10 could not be fitted. The extracted rate constants from the fitted data are summarized in Table S3.*

Table S3. Association and dissociation rate constants for Fabs from SPR data. *Association and dissociation rates were extracted from single exponential fits of the SPR data shown in Figure S7.*

		Association				Dissociation		
**Fab**	**[A] (M)**	**k (s^{-1})**	**k_{on} ($M^{-1}s^{-1}$)**	**95% CI**	**R^2**	**k_{off} (s^{-1})**	**95% CI**	**R^2**
**1**	5.00E-06	4.81E-02	9.62E+03	0.04717 to 0.04902	1.00	-	-	-
**2**	5.00E-06	-	-	-	-	-	-	-
**3**	1.00E-08	1.43E-02	1.43E+06	0.01415 to 0.01438	1.00	1.09E-03	0.001059 to 0.001130	1.00
**4**	5.00E-06	-	-	-	-	-	-	-
**5**	1.00E-08	6.16E-02	6.16E+06	0.05946 to 0.06389	0.99	7.15E-03	0.007038 to 0.007259	0.99
**6**	1.00E-08	9.61E-02	9.61E+06	0.09430 to 0.09785	1.00	9.50E-03	0.009388 to 0.009613	0.99
**7**	1.00E-08	1.09E-01	1.09E+07	0.1064 to 0.1123	0.99	3.24E-02	0.03210 to 0.03275	1.00

**8**	1.00E-08	3.29E-02	3.29E+06	0.03255 to 0.03327	1.00	1.38E-02	0.01370 to 0.01387	1.00
**9**	5.00E-06	2.99E-02	5.98E+03	0.02893 to 0.03081	0.99	1.64E-02	0.01620 to 0.01664	0.99
**10**	5.00E-06	-	-	-	-	-	-	-
**11**	5.00E-06	1.46E-01	2.92E+04	0.1388 to 0.1542	0.97	1.04E-01	0.09870 to 0.1105	0.84
**12**	1.00E-08	7.60E-02	7.60E+06	0.07484 to 0.07707	1.00	1.22E-02	0.01204 to 0.01228	1.00
**13**	1.00E-08	3.61E-02	3.61E+06	0.03579 to 0.03644	1.00	1.88E-02	0.01846 to 0.01910	0.99

5.2 Single-cycle SPR data for Fabs with rapid association and dissociation

Single-cycle SPR measurements were conducted using four different lactoferrin concentrations (1, 4, 16 and 64 nM) to validate the correctness of the determined association (k_{on}) and dissociation (k_{off}) rate constants, which were initially derived from measurements at a single lactoferrin concentration. This approach allowed for confirmation of the rate constants by ensuring consistency across varying concentrations and minimizing potential errors due to concentration-dependent effects.

As can be seen in Figure S8, the fit of the 1 nM data is less trustworthy due to the absence of a plateau within the measurement time. The fit of the 64 nM data is also less reliable due to saturation effects. The values obtained using 4 and 16 nM concentrations are within a good range, providing sufficient signal without saturation. These values match the results obtained in section 5.1, confirming their reliability (Tables S3 and S5).

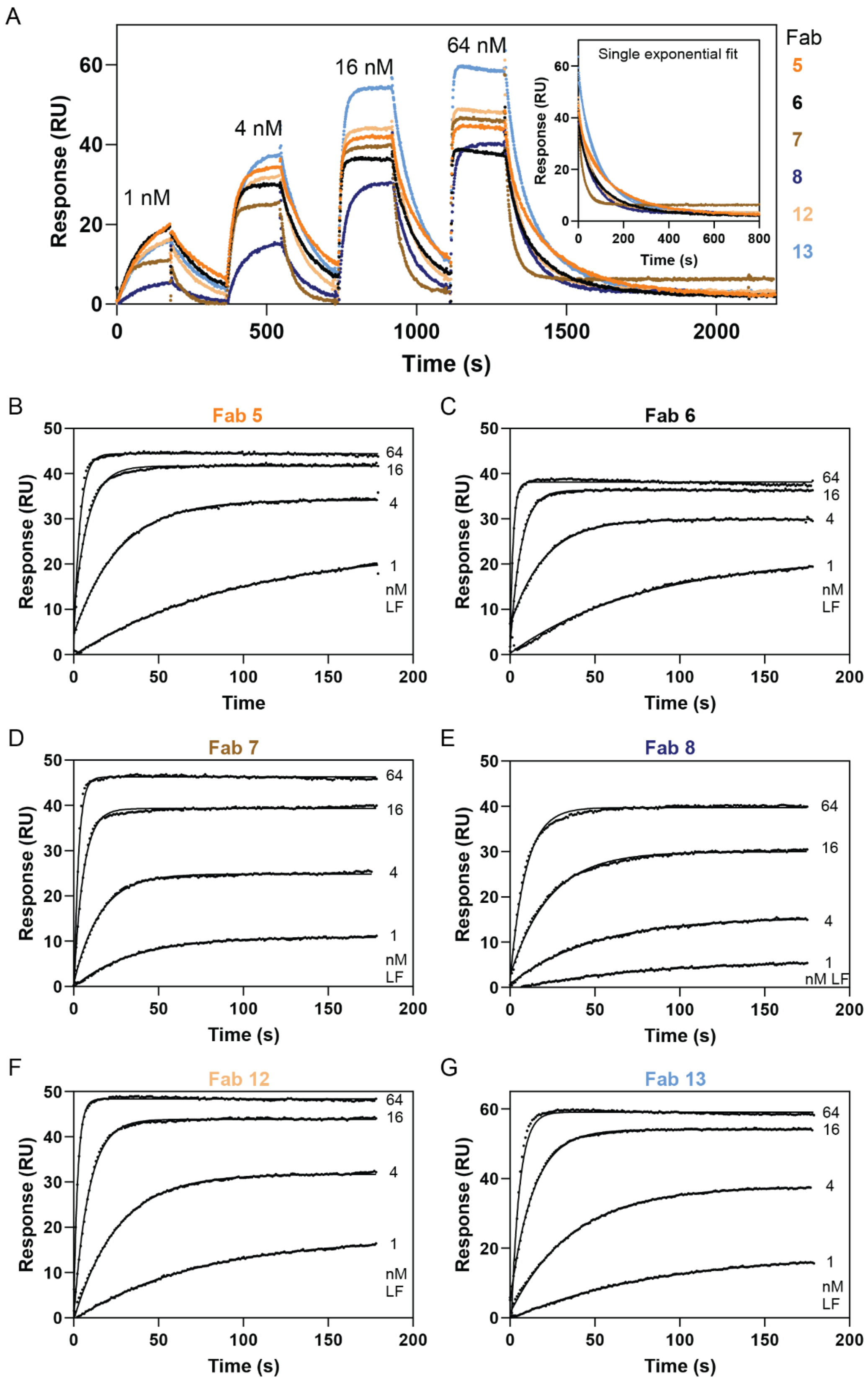

Figure S8. Single-cycle kinetic measurements for Fabs with rapid association and dissociation. *(A) Fabs 5, 6, 7, 8, 12, and 13 were immobilized on the SPR chip. For each Fab, four lactoferrin concentrations (1, 4, 16, and 64 nM) were applied. Association and dissociation were each measured for 180 s, followed by a 15-minute dissociation phase at the end. The 15-minute dissociation data were fitted using a single-exponential equation to extract the dissociation constants, which are shown in Table S4. (B–G) Association curves for each lactoferrin concentration for Fab 5 (B), 6 (C), 7 (D), 8 (E), 12 (F), and 13 (G). The data were fitted with a single-exponential equation, and the extracted parameters are presented in Table S5.*

Table S4. Dissociation constants extracted from single exponential fits in the inset of Figure S8A.

Fab	k_{off} (s^{-1})	95% CI	R^2
**5**	7.49E-03	0.007382 to 0.007594	0.992
**6**	1.01E-02	0.009984 to 0.01029	0.990
**7**	4.12E-02	0.04059 to 0.04172	0.991
**8**	1.45E-02	0.01446 to 0.01462	0.999
**12**	1.40E-02	0.01386 to 0.01423	0.992
**13**	1.06E-02	0.01043 to 0.01067	0.994

Table S5. Association parameters extracted from single exponential fits in Figure S8.

Fab	[A] (M)	k (s^{-1})	95% CI	k_{on} (M^{-1-s})	R^2
**5**	1.00E-09	0.010	0.0099 to 0.0105	1.02E+07	0.999
	4.00E-09	0.038	0.0375 to 0.0384	**9.49E+06**	0.999
	1.60E-08	0.121	0.1181 to 0.1237	**7.56E+06**	0.995
	6.40E-08	0.277	0.2648 to 0.2897	4.33E+06	0.976
**6**	1.00E-09	0.013	0.0121 to 0.0133	1.27E+07	0.992
	4.00E-09	0.050	0.0497 to 0.0507	**1.26E+07**	0.999
	1.60E-08	0.157	0.1549 to 0.1592	**9.81E+06**	0.998
	6.40E-08	0.558	0.5350 to 0.5817	8.71E+06	0.987
**7**	1.00E-09	0.029	0.0281 to 0.0293	2.87E+07	0.996
	4.00E-09	0.066	0.0644 to 0.0666	**1.64E+07**	0.997
	1.60E-08	0.177	0.1721 to 0.1812	**1.10E+07**	0.993
	6.40E-08	0.386	0.3682 to 0.4047	6.03E+06	0.976
**8**	1.00E-09	0.013	0.0120 to 0.0133	1.26E+07	0.992
	4.00E-09	0.020	0.0200 to 0.0209	**5.11E+06**	0.997
	1.60E-08	0.043	0.0422 to 0.0443	**2.71E+06**	0.995
	6.40E-08	0.108	0.1052 to 0.1117	1.69E+06	0.992
**12**	1.00E-09	0.014	0.0137 to 0.0142	1.39E+07	0.999
	4.00E-09	0.039	0.0385 to 0.0397	**9.78E+06**	0.998
	1.60E-08	0.113	0.1114 to 0.1143	**7.05E+06**	0.998
	6.40E-08	0.416	0.4064 to 0.4250	6.49E+06	0.996
**13**	1.00E-09	0.012	0.0116 to 0.0121	1.19E+07	0.999
	4.00E-09	0.027	0.0267 to 0.0278	**6.82E+06**	0.997
	1.60E-08	0.075	0.0745 to 0.0757	**4.69E+06**	0.999
	6.40E-08	0.208	0.1953 to 0.2205	3.24E+06	0.949

6. Continuous lactoferrin sensing using t-BPM

The fast-dissociating Fabs were integrated into the competition-based t-BPM sensor (Figure 4). Four lactoferrin concentrations (15.6, 62.5, 250, and 1000 nM) were tested with wash steps after each measurement to assess the binding reversibility (Figure 4B). All six Fabs showed a high bound fraction without free lactoferrin and a decrease when lactoferrin was added. This concentration series was measured multiple times for each Fab, shown in Figure S9. Signal drift was observed over time, which was corrected by normalizing the data to the baseline and sensor background, as is further explained in section 6.2.

6.1 Raw data Fab 5, 6, 7, 8, 12 and 13 in t-BPM

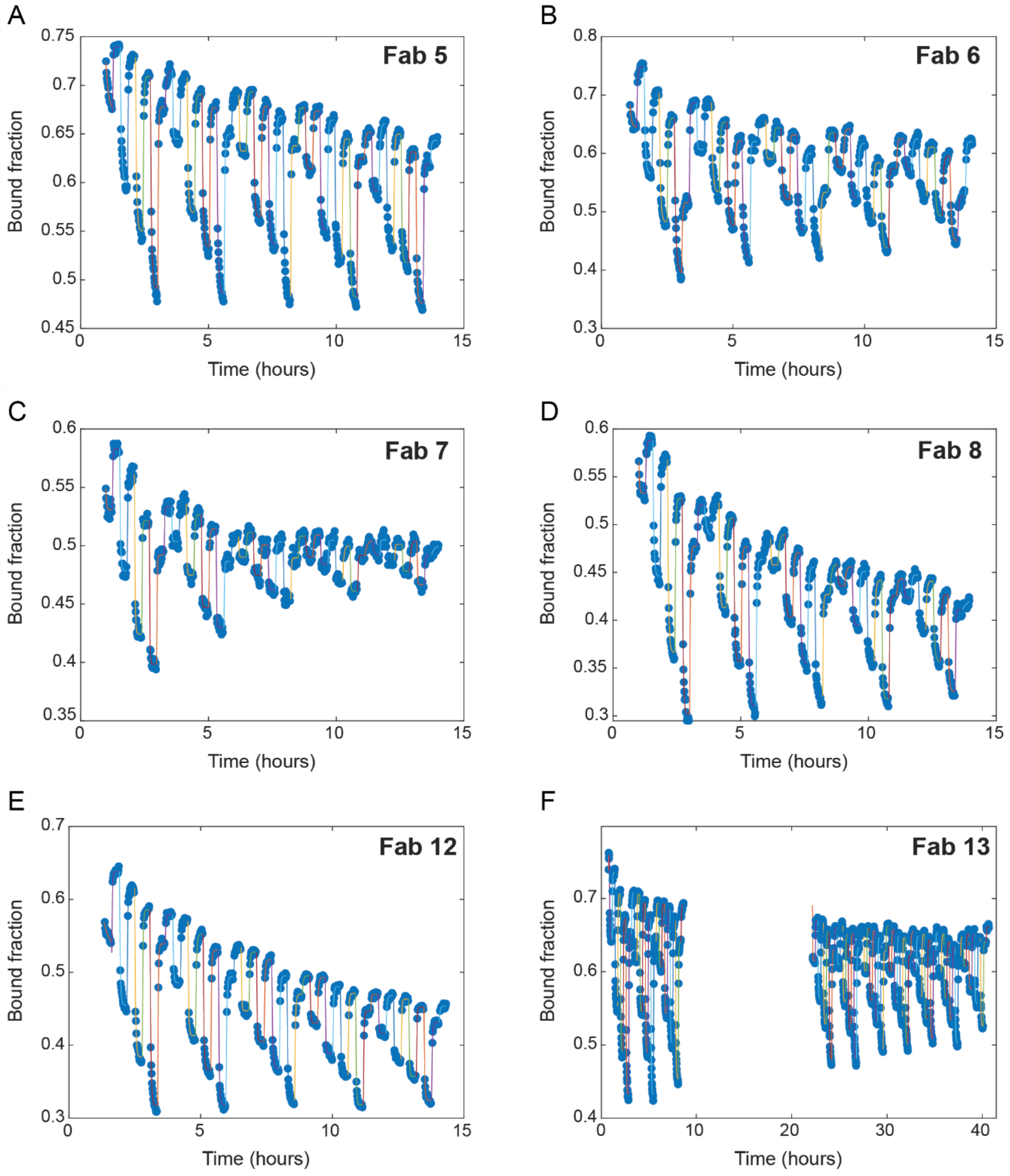

Figure S9. t-BPM sensor response over time at varying lactoferrin concentrations. *Six different Fabs were tested: Fab 5 (A), Fab 6 (B), Fab 7 (C), Fab 8 (D), Fab 12 (E), and Fab 13 (F). Samples were introduced at a flow rate of 100 µL/min for one minute, followed by measurement of the bound fraction during a no-flow phase at 1-minute intervals over 15 minutes. The bound fraction of each 1-minute measurement is represented by the blue dots and the colored lines are guides to the eye.*

6.2 Signal drift correction

To correct for signal drift over time, the data were normalized using linear interpolation between the highest and lowest signal points: the highest signal corresponds to the baseline measurement (in the absence of analyte), and the lowest signal corresponds to the background measurement (with a known high concentration of analyte). These points were used to create a linear reference line (Figure S10A and Table S6). Each data point in the time series was then adjusted according to its position relative to the reference line, effectively compensating for signal drift in the sensor (Equation S1). After normalization, a stable and reproducible signal was observed throughout the 40 hours (Figure S10B).

$$S_{normalized} = \frac{S - S_{BG}}{S_{BL} - S_{BG}} \quad \text{(Equation S1)}$$

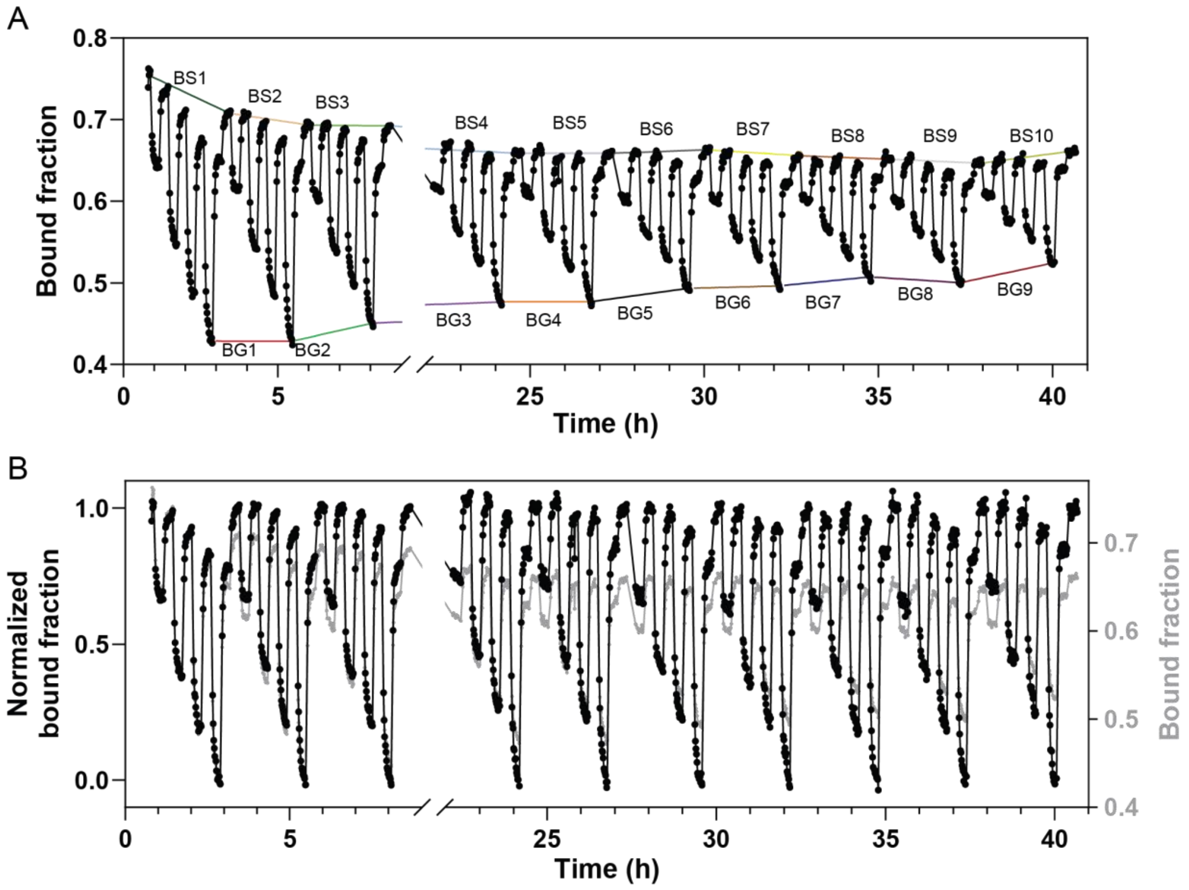

Figure S10. Signal drift correction using linear interpolation between the highest and lowest signal points. *(A) t-BPM sensor response using Fab 13. Black points represent the measurement data, connected by a solid black line. Solid colored lines indicate the linear fits between the background and baseline signals for each series. The equations for these fits are provided in Table S6. (B) The normalized sensor response is shown in black, while the uncorrected sensor response is displayed in grey.*

Table S6. Equations of the linear fit between background and baseline data points for each series.

**Background linear fits**		**Baseline linear fits**	
BG1	Y = -9.401e-005*X + 0.4290	BS1	Y = -0.01786*X + 0.7692
BG2	Y = 0.008421*X + 0.3826	BS2	Y = -0.005601*X + 0.7270
BG3	Y = 0.001623*X + 0.4374	BS3	Y = -0.0003147*X + 0.6951
BG4	Y = 5.221e-005*X + 0.4754	BS4	Y = -0.002094*X + 0.7105
BG5	Y = 0.005856*X + 0.3203	BS5	Y = 3.969e-005*X + 0.6577
BG6	Y = 0.001086*X + 0.4613	BS6	Y = 0.001517*X + 0.6173
BG7	Y = 0.004172*X + 0.3622	BS7	Y = -0.002724*X + 0.7450
BG8	Y = -0.002689*X + 0.6007	BS8	Y = -0.001663*X + 0.7103
BG9	Y = 0.009082*X + 0.1610	BS9	Y = -0.001899*X + 0.7186
		BS10	Y = 0.005685*X + 0.4308

6.3 Sensor response in buffer and milk samples

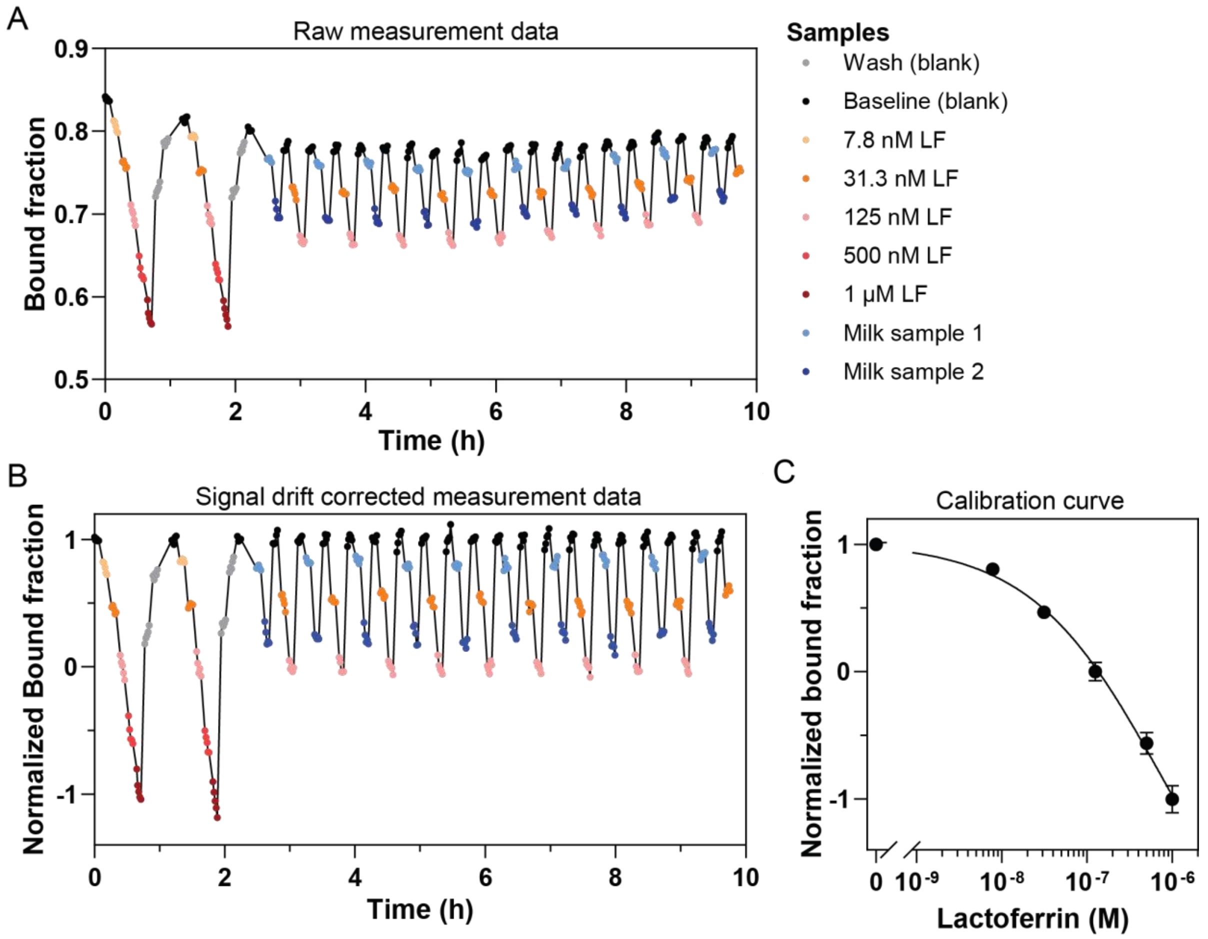

Figure S11. Lactoferrin measurements in buffer and milk samples using the competition-based t-BPM sensor. *(A) Measurement data as a function of time for different samples using Fab 13. First, two calibration series were measured using five samples of lactoferrin spiked in buffer (7.8, 31.3, 125, 500 and 1000 nM). Subsequently, buffer sample spiked with 31.3 nM LF (orange) and two milk samples (light and dark blue) were measured repeatedly over time. The milk samples were 10x diluted and centrifuged for 10 minutes at 10.000 rpm. The bound fraction was measured in the absence of flow with 1-minute intervals over a period of 5 minutes. (B) The measurement data was corrected using the baseline samples (black; 0 nM LF) and background samples (pink; 125 nM LF) as described in section 6.2. (C) The dose-response curve of the calibration samples was used to determine the concentrations corresponding to the signals measured for the buffer sample with 31.3 nM LF (orange) and the two milk samples (light and dark blue), which are presented in Figure 5C.*

6.4 Time dependencies in the t-BPM sensor response

After injection of a sample, the t-BPM sensor measures the bound fraction signal as a function of time by consecutive one-minute measurements. Figure S12A (corresponding to Figure 4B in the manuscript) shows the response for four sample injections alternated with four blank sample injections. The data show time-dependent responses, as indicated by the arrows. In this Section, we study if the time-dependent responses depend on analyte concentration and if the signal is stable when a sample injection is repeated.

Figure S12B shows the time-dependent responses for different analyte concentrations, including single-exponential fits, for a sensor with Fab 5. The graph shows that the time-dependent signals have indeed single-exponential behaviors. The fitted rate parameters for different analyte concentrations and Fabs are presented in Table S7 (post-lactoferrin injection) and Table S8 (after blank injection). The analysis reveals that the analyte concentration does not have a significant influence on the rate parameters.

After injection of the highest analyte concentration (1 µM LF), two consecutive blank sample injections were required to give a complete return to the baseline signal, see Figure S12A. In Figure S13, the effect of two sample injections is studied for all analyte concentrations (a single injection refers to 100 µL/min during one minute, so in total 100 µL per injection). The data show that the second flush has a significant effect only for the highest analyte concentration and not for the lower concentrations. So for the lower concentrations, the single-injection protocol is suitable for achieving sufficient fluid exchange.

These observations lead to the hypothesis that the time-dependencies in Figure S12A could be dominated by the effective dissociation rate constants of the binding between Fabs conjugated to the particles and LF conjugated to the surface. The origins of these time-dependencies will be further investigated in follow-up research.

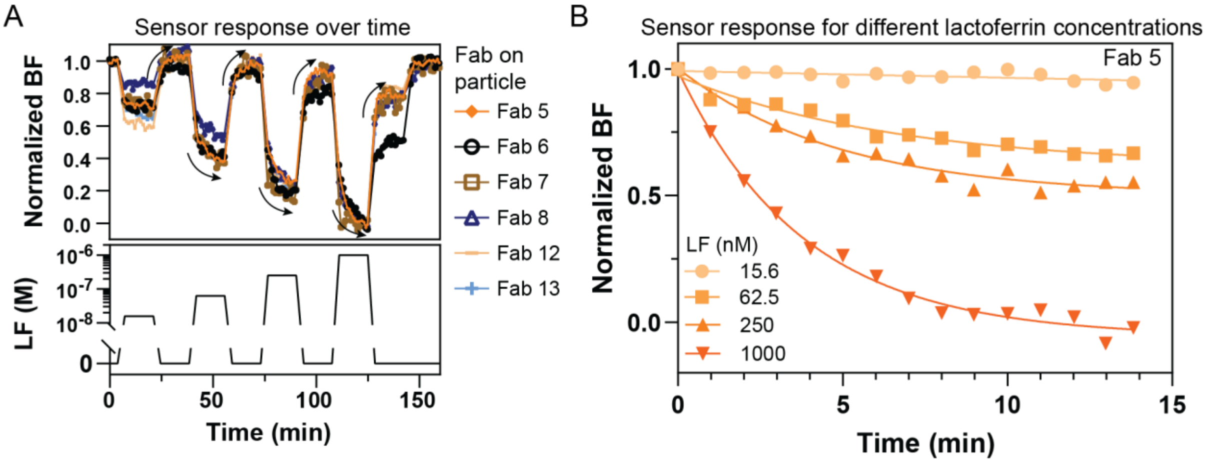

Figure S12. Analysis of time-dependency in the t-BPM sensor response. *(A) t-BPM sensor response over time at varying lactoferrin concentrations for different Fabs. The black arrows indicate observed time-dependent variations. (B) t-BPM sensor response for varying lactoferrin concentrations using Fab 5. Samples were flushed at 100 µL/min for one minute, and the bound fraction was measured at one-minute intervals for 15 minutes without flow. Solid lines represent single-exponential fits.*

Table S7. Signal rate parameters of the t-BPM sensor response after analyte addition. *Signal rates for three lactoferrin concentrations (62.5, 250, and 1000 nM) were obtained by fitting the signal response using a single-exponential fit (data from Figure S12A). An example of fitted data for Fab 5 is shown in Figure S12B.*

Fab	LF (nM)	Rate (min^{-1})	95% CI	R^2	Fab	LF (nM)	Rate (min^{-1})	95% CI	R^2
5	62.5	0.153	0.076 to 0.240	0.96	8	62.5	0.169	- to 0.469	0.79
	250	0.204	0.139 to 0.278	0.97		250	0.204	0.108 to 0.318	0.95
	1000	0.262	0.223 to 0.303	0.99		1000	0.155	0.080 to 0.240	0.97
6	62.5	0.764	0.432 to 1.352	0.91	12	62.5	0.576	0.287 to 1.044	0.87
	250	0.368	0.281 to 0.472	0.97		250	0.277	0.207 to 0.358	0.98
	1000	0.165	0.070 to 0.277	0.95		1000	0.271	0.231 to 0.315	0.99
7	62.5	0.104	- to 0.294	0.78	13	62.5	0.281	0.221 to 0.348	0.98
	250	0.231	- to 1.104	0.43		250	0.211	0.159 to 0.267	0.98
	1000	-	-	0.57		1000	0.282	0.242 to 0.326	0.99

Table S8. Signal rate parameters of the t-BPM sensor after blank addition. *A blank sample (buffer without analyte) was used to restore the signal to the baseline level and assess sensor reversibility. Signal rates were determined for four lactoferrin concentrations (15.6, 62.5, 250, and 1000 nM) by fitting the signal response using a single-exponential fit (data from Figure S12A).*

Fab	LF (nM)	Rate (min^{-1})	95% CI	R^2	Fab	LF (nM)	Rate (min^{-1})	95% CI	R^2
5	15.6	0.351	0.193 to 0.584	0.91	8	15.6	0.049	- to 0.207	0.82
	62.5	0.334	0.209 to 0.507	0.94		62.5	0.219	0.098 to 0.370	0.90
	250	0.645	0.435 to 0.984	0.92		250	0.350	0.124 to 0.748	0.87
	1000	1.895	0.951 to -	0.87		1000	0.406	0.020 to 1.358	0.76
6	15.6	1.043	0.406 to -	0.60	12	15.6	0.625	0.471 to 0.827	0.96
	62.5	1.348	0.586 to -	0.77		62.5	0.262	0.137 to 0.428	0.92
	250	0.251	- to 1.233	0.68		250	0.603	0.392 to 0.917	0.94
	1000	0.232	0.067 to 0.460	0.81		1000	0.547	0.416 to 0.717	0.96
7	15.6	-	-	0.19	13	15.6	0.517	0.350 to 0.756	0.92
	62.5	0.721	0.187 to -	0.47		62.5	0.450	0.341 to 0.588	0.97
	250	0.288	0.022 to 1.183	0.66		250	0.391	0.256 to 0.577	0.95
	1000	-	-	0.38		1000	0.505	0.384 to 0.660	0.97

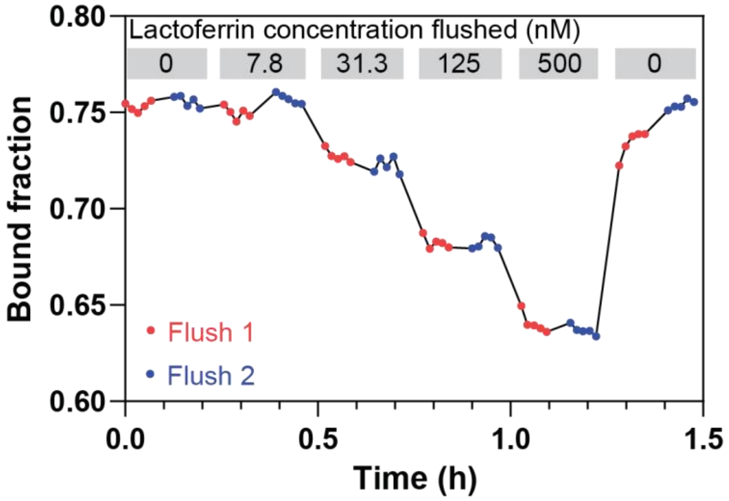

Figure S13. Effect of repeated flushing on t-BPM sensor response. *t-BPM sensor response for Fab 13 at varying lactoferrin concentrations. Each sample was flushed at 100 µL/min for one minute and measured five times consecutively. This procedure was repeated twice for each sample (red = first flush/measurement, blue = second flush/measurement). The lactoferrin concentrations are indicated in the grey boxes above the data.*

References

(1) Bergkamp, M.H.; Cajigas, S.;van IJzendoorn, L.J.;Prins, M. W. J. High-Throughput Single-Molecule Sensors: How Can the Signals Be Analyzed in Real Time for Achieving Real-Time Continuous Biosensing. *ACS Sensors* **2023**. https://doi.org/10.1021/acssensors.3c00245.

(2) Buskermolen, A. D.; Lin, Y.-T.; van Smeden, L.; van Haaften, R. B.; Yan, J.; Sergelen, K.; de Jong, A. M.; Prins, M. W. J. Continuous Biomarker Monitoring with Single Molecule Resolution by Measuring Free Particle Motion. *Nat. Commun. 2022 131* **2022**, *13* (1), 1–12. https://doi.org/10.1038/s41467-022-33487-3.

(3) Visser, E. W. A.; Yan, J.; Van IJzendoorn, L. J.; Prins, M. W. J. Continuous Biomarker Monitoring by Particle Mobility Sensing with Single Molecule Resolution. *Nat. Commun.* **2018**, *9* (1). https://doi.org/10.1038/s41467-018-04802-8.

(4) Lin, Y.-T.; Vermaas, R.; Yan, J.; M. de Jong, A.; W.J. Prins, M. Click-Coupling to Electrostatically Grafted Polymers Greatly Improves the Stability of a Continuous Monitoring Sensor with Single-Molecule Resolution. *ACS Sensors* **2021**, *6* (5), 1980–1986. https://doi.org/10.1021/acssensors.1c00564.

(5) van Smeden, L. *Continuous Monitoring of Health Markers : A Study on BPM Immunoassays and Microdialysis,* 2023, Eindhoven University of Technology.

(6) Vu, C.; Yan, J.; de Jong, A. M.; Prins, M. W. J. How Highly Heterogeneous Sensors with Single-Molecule Resolution Can Result in Robust Continuous Monitoring Over Long Time Spans. *Adv. Sci.* **2024**, *2412181*, 1–9. https://doi.org/10.1002/advs.202412181.

(7) Yan, J.; van Smeden, L.; Merkx, M.; Zijlstra, P.; W. J. Prins, M. Continuous Small-Molecule Monitoring with a Digital Single-Particle Switch. *ACS Sensors* **2020**, *5* (4), 1168–1176. https://doi.org/10.1021/acssensors.0c00220.

(8) Keeble, A. H.; Turkki, P.; Stokes, S.; Khairil, I. N. A.; Rahikainen, R. Approaching Infinite Affinity through Engineering of Peptide – Protein Interaction. **2019**, No. 13. https://doi.org/10.1073/pnas.1909653116.
